# Antimicrobial and Cytotoxic Activities of Biogenic Nanoparticles Produced by Cyanobacteria

**DOI:** 10.1002/cbic.202500882

**Published:** 2026-02-25

**Authors:** Laíne Santos Ribeiro, Rhuana Valdetário Médice, Janaína Morone Bavini, Camila Manoel Crnkovic, Samuel Cavalcante do Amaral

**Affiliations:** ^1^ Escola de Artes Ciências e Humanidades Universidade de São Paulo São Paulo Brazil; ^2^ Faculdade de Ciências Farmacêuticas Universidade de São Paulo São Paulo Brazil; ^3^ CIIMAR—Interdisciplinary Centre of Marine and Environmental Research University of Porto Matosinhos Portugal

**Keywords:** antimicrobial, biogenic nanoparticles, biotechnology, cyanobacteria, cytotoxic activity

## Abstract

Biogenic nanoparticles are distinguished by their unique physical and chemical attributes, notably their potent antimicrobial activity against bacterial and fungal pathogens, as well as their cytotoxic effects on cancer cells. These nanoparticles are characterized by their biocompatibility, indicating their potential as effective antimicrobial agents and in oncological therapies. This article examines the existing literature on the antimicrobial and cytotoxic properties of nanoparticles derived from cyanobacteria, with particular emphasis on their implications for human health.

## Graphical abstract

The article reviews the green synthesis of biogenic nanoparticles produced by cyanobacteria, highlighting their antimicrobial and cytotoxic properties. These nanoparticles, formed intra‐ or extracellularly, show activity against Gram‐positive and Gram‐negative bacteria as well as fungi and can induce apoptosis in tumor cells. With biocompatibility and structural diversity, they emerge as promising alternatives in biotechnology and cancer therapies.

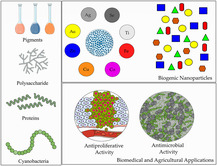

## 1 Introduction

In recent years, the application of nanotechnology has been extensively explored across various scientific fields [1, 2]. The term “nano” refers to particles or materials with dimensions in the range of 1–100 nm [3, 4]. Nanoparticles (NPs) are characterized by their high surface area‐to‐volume ratio, which leads to notable variations in catalytic and thermal properties, melting points, conductivity, mechanical strength, and optical absorption [5].

There are several approaches to producing NPs with diverse shapes and sizes, including conventional physicochemical methods and modern techniques, such as green synthesis [5]. In chemical synthesis, two main strategies are employed: the top‐down approach, which involves the breakdown or deconstruction of bulk materials, and the bottom‐up approach, in which atom‐by‐atom or molecule‐by‐molecule nucleation occurs to form NPs using reducing agents, precursors, and stabilizers. Common reducing agents include ascorbate, sodium borohydride, and sodium citrate, while stabilizers often comprise sodium carboxylate, starch, methylcellulose, and polyvinylpyrrolidone. Physicochemical approaches frequently encounter challenges related to slow production rates, high energy requirements, and escalating costs [6].

Green chemistry has emerged as an innovative and effective approach to addressing the challenges associated with traditional nanomaterial production. This methodology promotes sustainability by minimizing waste, reducing energy consumption, and limiting the use of hazardous precursors. It emphasizes the importance of replacing toxic compounds and solvents with safer alternatives, optimizing synthesis conditions to conserve both reagents and energy, and implementing environmentally friendly parameters, such as mild pH, temperature, and pressure. This strategic focus on sustainability not only enhances environmental protection but also contributes to the development of safer, more efficient nanomaterials [3].

Plant extracts and various microorganisms, such as fungi, bacteria, microalgae, and yeasts, are extensively utilized in the synthesis of biological NPs. These systems produce a diverse array of biomolecules, including proteins, enzymes, and pigments, as well as compounds like phycobiliproteins, extracellular polysaccharides, phenolic compounds, alkaloids, and fatty acids [5]. Furthermore, these microorganisms possess a wide range of secondary metabolites that can further enhance the biological properties of NPs. In particular, the bioactive compounds derived from cyanobacteria offer notable benefits for biomedical and cosmeceutical applications. Their antioxidant, anti‐inflammatory, and photoprotective effects contribute to improved functionality of biosynthesized NPs [7].

In this context, cyanobacteria are recognized as a promising source for the production of NPs that possess unique and valuable properties [5]. These microorganisms are the oldest and most widely distributed photosynthetic prokaryotic autotrophs, exhibiting remarkable diversity in morphology, pigment composition, and natural product synthesis [8]. Many cyanobacterial species and their extracts can interact with metal precursors, enabling the environmentally friendly synthesis of NPs. This process can occur either intracellularly or extracellularly, employing a variety of metals such as gold, silver, copper, iron, zinc, selenium, and titanium [9, 10, 11, 12, 13].

The rising issue of bacterial resistance, mainly caused by the overuse of antibiotics, has become a significant public health concern. NPs have proven effective against both Gram‐negative and Gram‐positive bacteria due to their small size and high surface area‐to‐volume ratio [14]. In addition to their antimicrobial potential, NPs have gained considerable attention in cancer research. Their ability to selectively target tumor cells, deliver therapeutic agents, and induce cytotoxic effects offers promising strategies for oncological treatments. In particular, biosynthesized NPs are emerging as versatile candidates for advanced biomedical applications, exhibiting potent antimicrobial and anticancer activities attributable to their physicochemical characteristics and diverse mechanisms of action [15].

Some reviews have examined the application of NPs derived from cyanobacteria [5]. However, many of these studies have primarily focused on NPs produced only from a metal precursor [16, 17, 18] or on the fabrication processes. These studies often focus on general biotechnological applications without providing detailed insights into specific biological activities. Therefore, this review aims to comprehensively discuss the synthesis and characterization of cyanobacterial NPs while also critically evaluating their biological activities, with particular emphasis on antimicrobial and cytotoxic properties. The analysis includes studies retrieved from PubMed, ScienceDirect, Scopus, and Web of Science dated from 2015 to 2025. The search employed the keywords “Cyanobacteria,” “Nanoparticles,” “Antimicrobial,” “Antibacterial,” “Antifungal,” and “Cytotoxicity.” In this context, antimicrobial NPs were defined as those capable of inhibiting the growth of bacteria and fungi, while cytotoxicity assessments were limited to studies involving mammalian cell lines.

## 2 Modes of Synthesis of Biogenic NPs

The green synthesis of NPs is achieved using biological materials derived from microorganisms, plants, or algae. This approach is more environmentally friendly than traditional chemical methods because it often simplifies the synthesis process, requires less specialized equipment, and reduces the generation of hazardous by‐products. NPs can be produced directly from the living cells due to the presence of biological molecules that act as coating agents [19].

Cyanobacteria are a promising alternative for NP synthesis due to their ability to survive in extreme environments and their biomolecules and molecular mechanisms [20, 21]. Furthermore, the chemical diversity of the phylum ensures the production of NPs with diverse properties and potential applications. Polysaccharides and proteins act as reducing agents to produce NPs in cyanobacteria, which can be produced either intracellularly or extracellularly [16].

**FIGURE 1:**
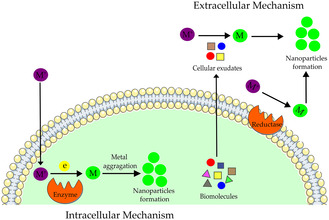
Intracellular and extracellular pathways involved in the biosynthesis of metal nanoparticles. In the intracellular pathway, metal ions are transported into the cell, where intracellular enzymes and metabolites reduce them, leading to nanoparticle nucleation and formation within the cytoplasm or associated cellular compartments. In the extracellular pathway, secreted or released biomolecules in the culture supernatant function as reducing and stabilizing agents, facilitating metal ion reduction and NPs formation outside the cell.

In extracellular synthesis, NPs are produced outside the cell using biomolecules secreted into the surrounding medium. These biomolecules, which include water soluble exopolysaccharides (EPSs), enzymes, proteins, and pigments, serve as reducing and stabilizing agents, converting metal ions into NPs (Figure 1) [22]. Nitrate reductase is one example of an enzyme involved in these processes. In *Bacillus subtilis* EWP‐46, it may facilitate silver NP (AgNP) synthesis by promoting nitrate ion production and reducing silver ions [23]. This reduction likely occurs through electron transfer from NADH, with NADH‐dependent reductase acting as the electron carrier [24]. In the case of proteins, these macromolecules are normally located on the surface of NPs. Their functional groups —OH, —NH_2_, —SH_2_, —CHO, and —COOH were found to contribute to the reduction of silver nitrate and the stabilization of AgNPs [25, 26]. The synthesis of NPs by EPSs is attributed to their chelating capacity, which facilitates the binding of metal ions, followed by reduction and stabilization via electrostatic interactions. The polymeric structure of EPSs also forms a network via hydrogen bonding, which can stabilize NPs [27]. For pigments such as carotenoids, phycocyanin, and phycoerythrin released into the medium, the functional hydroxyl groups actively participate in the NPs’ production [28]. The proposed mechanism for carotenoids and chlorophyll involves a redox reaction, in which the electron donor undergoes oxidation in the presence of metallic salts in solution [29].

In intracellular synthesis, NPs are generated within cells and must subsequently be extracted for application. The reducing agents found in the organisms, such as pigments, enzymes, peptides, and polysaccharides, are located within the cell [19]. Figure 1 illustrates the two primary biosynthetic pathways in cyanobacteria: extracellular synthesis, in which NPs are produced outside the cell via secreted biomolecules, and intracellular synthesis, which occurs within the cellular matrix.

The NP size is influenced by the dimensions of the cell, including the cell wall and other specific components. This process involves internal absorption, which depends on cellular metabolism and is directed to cytoplasmic organelles and other cellular components [30].

In cyanobacteria, the thylakoid membrane plays a crucial role in intracellular synthesis by mediating ion transport through photosynthetic electron transport channels [16]. In this process, metal ions move within the cells, are transformed into NPs, and are extracted from the host cells using physical methods [19].

In both methods, several physical parameters, including pH, aeration, temperature, incubation time, and metal salt concentration, directly affect the shape and size of NPs. Initial confirmation of the synthesis is performed using ultraviolet–visible (UV–Vis) spectroscopy. This is followed by detailed analyses employing microscopic and analytical techniques such as scanning electron microscopy, transmission electron microscopy, X‐ray diffraction, and Fourier transform infrared (FTIR) spectroscopy [19].

## 3 Mechanism of Action of Biogenic NPs

The antimicrobial activity of NPs is mainly linked to the generation of free radicals on their surfaces. NPs can efficiently bind to microbial cell walls, leading to electrostatic interactions with the cell membrane [31]. This interaction induces the formation of reactive oxygen species (ROS), including hydroxyl radicals, superoxide anions, and hydrogen peroxide [32]. These ROS can oxidize cellular components, damaging proteins and nucleic acids, resulting in membrane disruption, enzyme inactivation, and further damage to DNA, RNA, and proteins [13]. Additionally, ROS can interact with the electron transport chain in microbial cells, impairing energy production and causing cell death [33]. In some cases, such as with gold, silver, and copper NPs, physical damage to the cell wall, including pitting and deformities, has been observed, which further disrupts cellular respiration and other vital processes [10, 34, 35].

Another important mechanism involves the release of metal ions from NPs into the surrounding microenvironment. Ions such as Zn^2+^, Ag^+^, Cu^2+^, and Se^2−^ can bind to protein functional groups, leading to protein denaturation and enzyme inactivation [34, 36, 37]. Furthermore, these ions may interfere with protein synthesis, DNA replication, and RNA integrity, disrupting essential cellular processes [38, 39]. In the case of copper NPs, Cu^2+^ ions have been shown to degrade the helical structure of DNA, cause genetic fragmentation, and induce mitochondrial dysfunction, ultimately triggering apoptosis [10, 40].

The antifungal activity of NPs is mediated through similar mechanisms, particularly the generation of ROS and the induction of oxidative stress [12, 13, 41]. These effects may result in pore formation in the fungal membrane, alterations in lipid composition, cytochrome c release, and activation of caspase cascades, thereby promoting apoptosis. NPs may also interfere with the activity of periplasmic enzymes, dehydrogenases, and active transport systems, in addition to inhibiting protein and nucleic acid synthesis, ultimately compromising fungal cell viability [12, 34].

**FIGURE 2:**
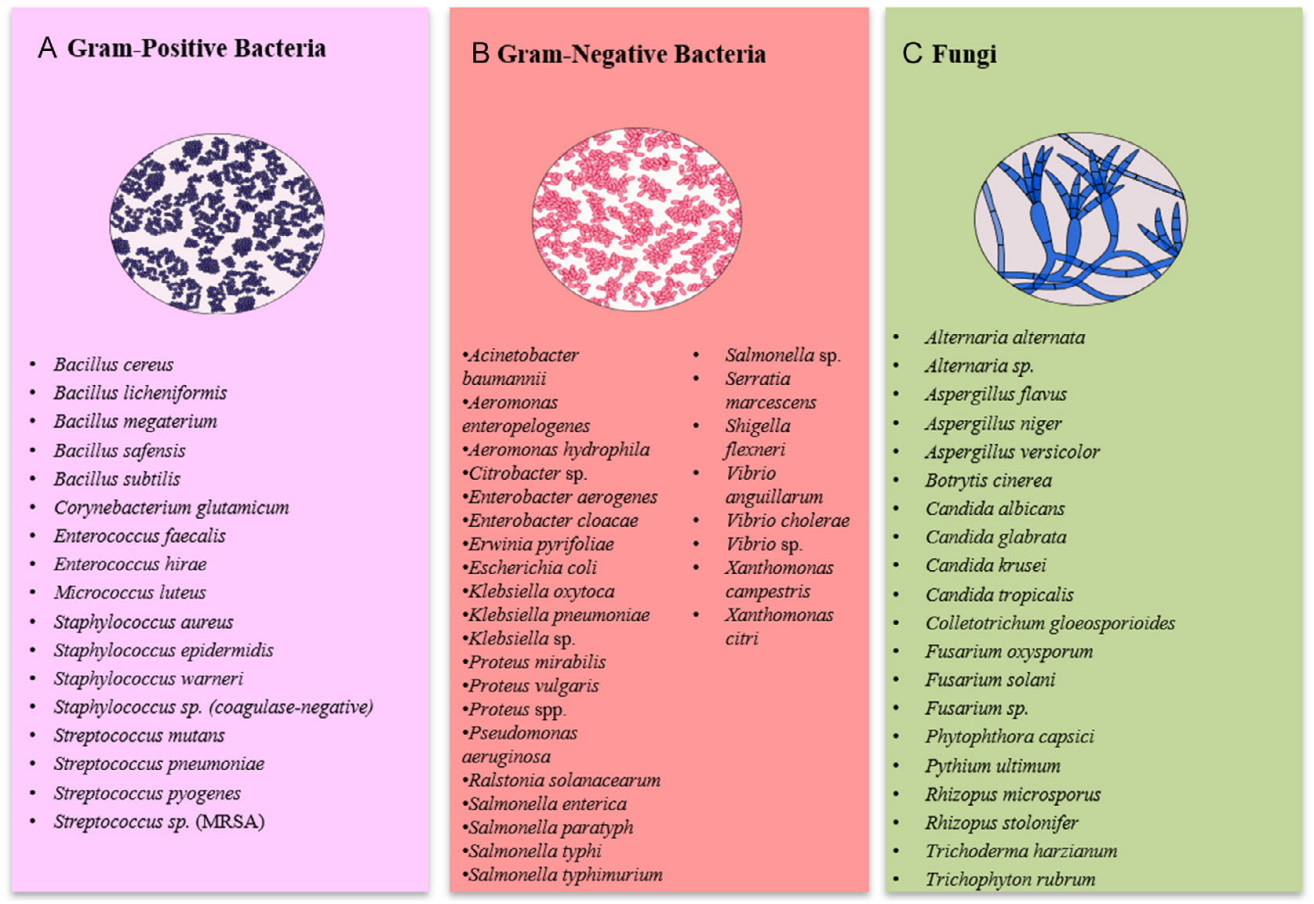
Main microbial targets of biogenic NPs produced by cyanobacteria.

Several factors, such as NP size and specific surface area, also directly influence antimicrobial and antifungal efficacy. In general, NPs of smaller dimensions tend to exhibit higher antimicrobial activity and greater in vitro toxicity, whereas larger particles often require higher concentrations to achieve equivalent effects. For example, AgNPs with diameters around ∼10–15 nm showed larger inhibition zones [42], and selenium NPs of 6.4–15.8 nm also displayed notable activity [43], while preparations containing substantially larger particles (100–200 nm) presented less potent antimicrobial effects under comparable conditions [44, 45]. This trend is observed across different metals and biological matrices—for instance, *Spirulina*‐derived NPs in the 7.8–18 nm range [46] produced stronger antimicrobial responses than some formulations with larger particles [47]. Smaller NPs present a greater contact area with microorganisms, facilitating increased metal ion release and enhancing toxic effects [48]. Another relevant aspect is the difference in susceptibility between Gram‐positive and Gram‐negative bacteria. Generally, Gram‐positive bacteria tend to be more susceptible to certain NPs, such as selenium‐based NPs, due to their lower levels of lipopolysaccharides in their cell walls, which reduce electrostatic repulsion and facilitate NP adhesion (Figure 2) [49].

The mechanism of action of silver NPs (AgNPs) against MCF‐7 cells primarily involves oxidative stress, increased ROS production, mitochondrial dysfunction, enzyme denaturation, and the disruption of cellular homeostasis, including ATPase inhibition [50]. Cytomorphological changes include plasma membrane damage, reduced microvilli, mitochondrial swelling, and chromatin condensation, suggesting apoptosis via mitochondrial pathways and p53 activation. AgNPs also act as photothermal agents, inducing hyperthermia and inhibiting cell proliferation [51]. Biogenic gold NPs (AuNPs) exert cytotoxic effects by disrupting the cell cycle in the S phase and promoting cell accumulation in the G_0_ phase [52]. Selenium NPs (SeNPs), in turn, can cross ion channels and directly interact with DNA and intracellular proteins, leading to DNA fragmentation, mitochondrial dysfunction, and apoptosis induction [49].

## 4 Antimicrobial and Cytotoxic NPs From Cyanobacteria

**FIGURE 3:**
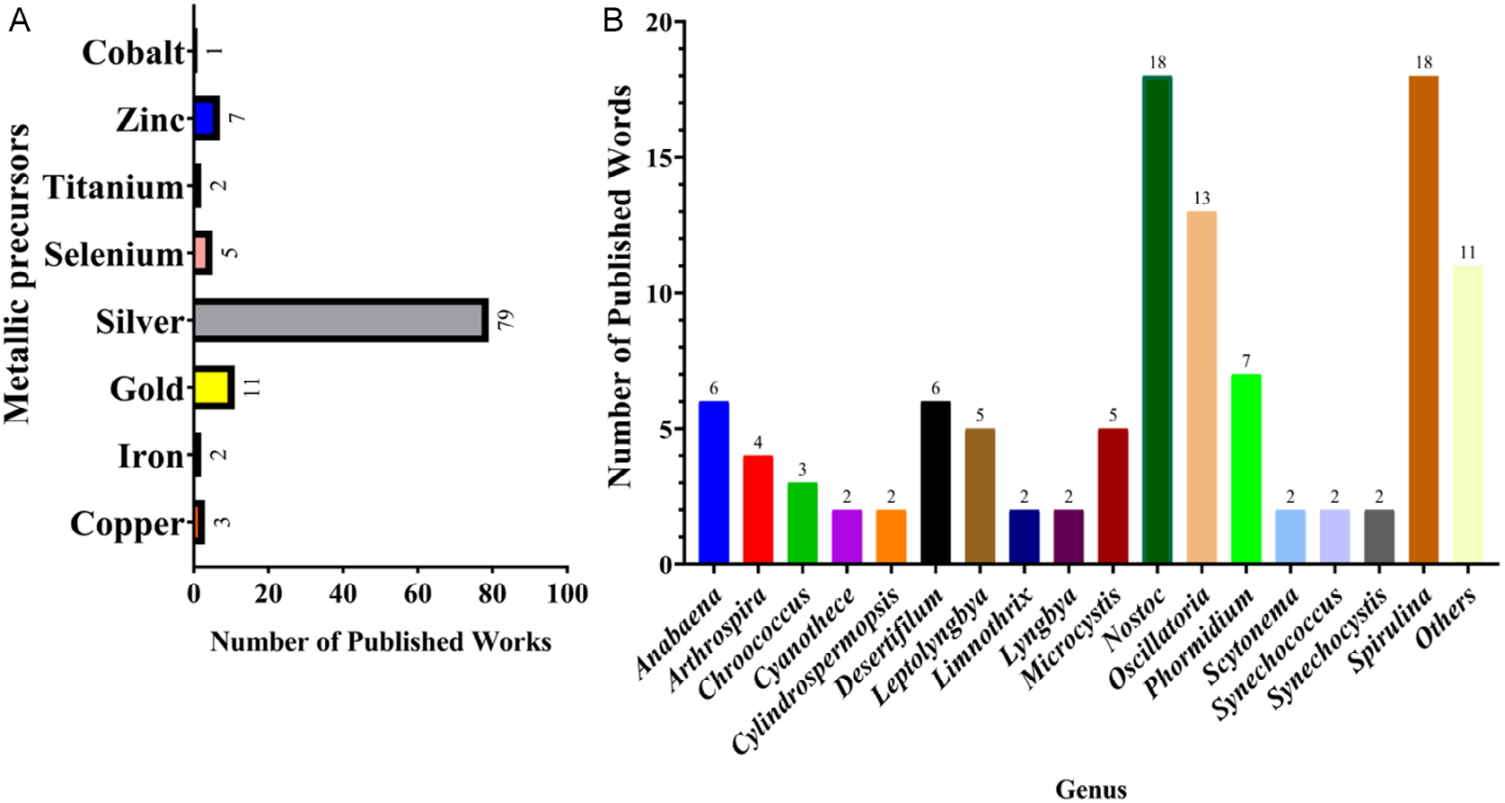
Distribution of key parameters related to nanoparticle (NP) biosynthesis mediated by cyanobacteria based on published studies from 2015 to 2025. (A) Number of studies reporting the use of metallic precursors for NP production employing cyanobacteria. Studies investigating more than one metallic precursor were counted multiple times, once for each precursor reported. (B) Frequency of cyanobacterial genera employed in NP synthesis studies. Articles reporting more than one cyanobacterial genus were counted multiple times, according to each genus investigated. “Others” refers to cyanobacterial genera with only one documented study: *Acacia*, *Aliinostoc*, *Characium*, *Coleofasciculus*, *Nodosilinea*, *Nodularia*, *Oxynema*, *Phormidesmis*, *Plectonema*, *Pseudanabaena*, and *Trichodesmium*.

Cyanobacteria have been widely investigated for their capacity to biosynthesize various types of NPs, including metallic NPs—such as silver and gold [53, 54], metalloids, such as selenium [55], and metal oxides, including iron, copper, zinc, and titanium oxides [13, 40, 56, 57]. Figure 3A illustrates the main metal precursor employed in the production of NPs derived from cyanobacteria. It is important to note that this distribution does not cover the full range of applications, as cyanobacteria‐based NPs have also been studied in other fields, including biosensing and medical imaging [16]. Research into the antimicrobial and cytotoxic properties of NPs originating from cyanobacteria has mainly focused on those specimens capable of forming filaments, mainly *Nostoc* and *Spirulina* (Figure 3B) [10]. A similar phenomenon has also been observed in the research of natural products [20, 58]. Filamentous cyanobacteria typically have larger genomes than their unicellular counterparts. This genomic diversity is often associated with a greater number of biosynthetic gene clusters, which are responsible for producing biologically active compounds [59].

**FIGURE 4:**
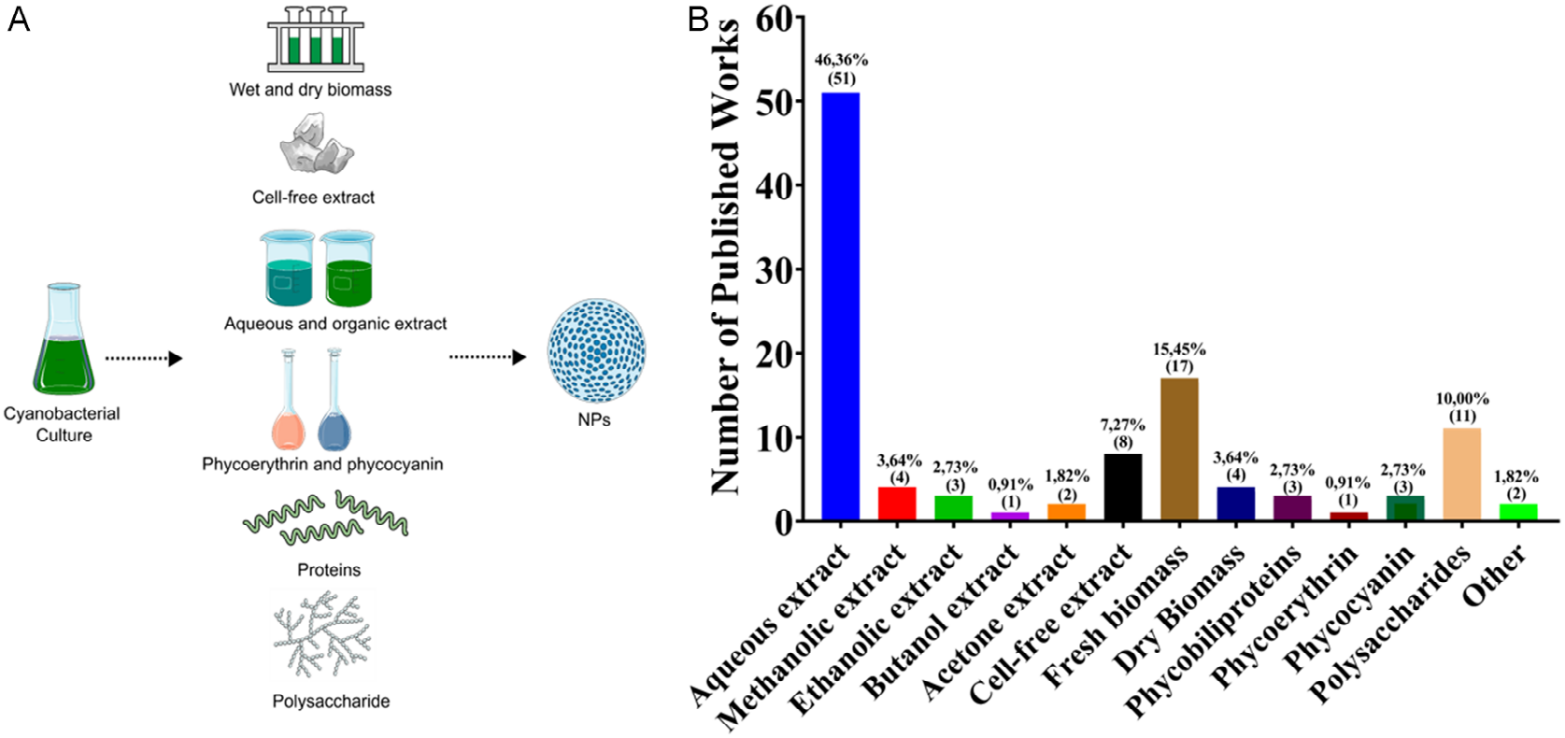
Cyanobacteria‐derived materials used in nanoparticle (NP) biosynthesis. (A) Commonly employed cyanobacterial materials, including wet and dry biomass, cell‐free extracts, and isolated biomolecular fractions. (B) Distribution of published studies reporting the use of different cyanobacteria‐derived materials for NP production. Studies reporting multiple materials were counted independently for each category.

Various strategies for producing NPs using cyanobacteria have been explored, which can be broadly classified into two main approaches [60]. The first approach is biosynthesis, where live cyanobacterial cells actively facilitate the formation of NPs through their metabolic processes. In contrast, the second approach utilizes materials derived from cyanobacteria, including cell extracts and EPSs, to synthesize NPs under controlled laboratory conditions (Figure 4A). Furthermore, within the second approach, fresh biomass and EPSs are also commonly utilized, emphasizing their role as natural reservoirs of bioactive compounds (Figure 4B). Besides these, cell‐free extracts, methanolic extracts, and phycobiliproteins have been used to a moderate degree. Taken together, these findings highlight the diversity of biological materials used in NP synthesis and indicate a preference for aqueous extractions, likely due to their simplicity, low toxicity, and biocompatibility (Figure 4B) [19].

## 5 Antimicrobial Activities of Different Metallic NPs

### 5.1 Iron Oxide NPs (IONPs)

Iron oxide NPs (IONPs) have attracted growing interest in biomedicine, agriculture, and environmental science due to their unique properties. These include simple separation methods, superparamagnetic behavior, high surface area, and greater surface‐to‐volume ratio. IONPs also exhibit biocompatibility and low toxicity. Combined with magnetic properties, these characteristics make IONPs suitable for drug delivery targeting organs and tumors [17].

Aqueous extracts from cyanobacteria have demonstrated the presence of chemical groups, such as aldehyde, sulfate, and hydroxyl, capable of reducing ferric ions into IONPs with antimicrobial activity. These IONPs exhibit a size range of 21–84 nm and a trigonal shape (Table 1) [13, 40].

**TABLE 1:** Antimicrobial activity of cyanobacteria‐mediated nanoparticles reported in the literature.

Cyanobacteria	Biocomponents	Metal precursor	Size, nm	Shape	Target	Activity (MIC; ZOI)	Reference
*Oscillatoria* sp.	Methanolic extract	Silver	10	Spherical	*Bacillus cereus*	7–17 mm	[42]
					*Citrobacter* sp.	11–18 mm	
					*E. coli* ATCC 11775	13–15 mm	
					*E. coli* ATCC 35218	6–20 mm	
					*P. aeruginosa* ATCC 27853	6–15 mm	
					*S. aureus* ATCC 29213	7–13 mm	
					*S. typhi* ATCC 14028	7–14 mm	
*Anabaena variabilis* NCCU‐441	Aqueous extract	Selenium	6.4–15.8	Spherical	*B. subtilis*	7.5–10.5 mm	[43]
					*C. albicans*	7.2–10 mm	
					*Candida glabrata*	6.5–11 mm	
					*Candida krusei*	7.5–12 mm	
					*E. coli*	7.5–5 mm	
					*K. pneumoniae*	7–9 mm	
					*S. aureus*	7–10 mm	
*Arthrospira indica* SOSA‐4	Aqueous extract	Selenium	4.5–14	Spherical	*C. albicans* ATCC 90028	300 μg/mL; 15.83 mm	[61]
					*Candida glabrata* ATCC 90030	400 μg/mL; 10.5 mm	
					*Candida tropicalis* ATCC 750	300 μg/mL; 11.67 mm	
					*E. coli* MTCC 443	8 μg/mL; 10.03–14.03 mm	
					*S. aureus* MTCC 902	16 μg/mL; 7.33–12.13 mm	
*Anabaena variabilis*	Aqueous extract	Silver	10–15	Spherical	*Bacillus cereus* MCC 2243	25 μg/L	[41]
					*E. coli* MCC 2412	12.5 μg/mL	
					*K. pneumoniae* KJ 938546	12.5 μg/mL	
					*P. aeruginosa* MTCC 2453	6.25 μg/mL	
*Spirulina platensis*	Methanolic extract	Zinc	8–31	Hexagonal	*K. pneumoniae*	62.5 μg/mL; 11.43–14.57 mm	[62]
					*S. aureus*	31.25 μg/mL; 15.47–17.33 mm	
					*S. pyogenes*	13.53–14.57 mm	
					*S. typhi*	10.47–12.57 mm	
*Cyanothece‐*like sp.	Fresh biomass	Silver	34–92	Angular	*Erwinia pyrifoliae*	6–10 mm	[48]
					*Staphylococcus warneri*	10–17 mm	
					*Xanthomonas citri*	9–10 cm	
*Spirulina platensis*	Aqueous extract	Silver	7.75–18.05	Spherical	*Aspergillus niger* NRC 53	17 mm	[46]
					*B. subtilis* ATCC 6633	No effect	
					*C. albicans*	22 mm	
					*Candida tropicalis* ATCC 750	15 mm	
					*E. coli* ATCC 25922	No effect	
					*Fusarium solani* NRC 15	15 mm	
					*S. aureus* ATCC 29213	No effect	
					*Salmonella enterica* ATCC 25566	No effect	
*Spirulina platensis*	Ethanolic extract	Silver	7.75–18.05	Spherical	*Aspergillus niger* NRC 53	28 mm	[46]
					*B. subtilis* ATCC 6633	24 mm	
					*C. albicans*	30 mm	
					*Candida tropicalis* ATCC 750	28 mm	
					*E. coli* ATCC 25922	22 mm	
					*Fusarium solani* NRC 15	25 mm	
					*S. aureus* ATCC 29213	22 mm	
					*Salmonella enterica* ATCC 25566	25 mm	
*Spirulina platensis*	Dry biomass	Silver	7.75–18.05	Spherical	*B. subtilis* ATCC 6633	20 mm	[46]
					*Aspergillus niger* NRC53	20 mm	
					*C. albicans ATCC* 10321	25 mm	
					*Candida tropicalis* ATCC 750	25 mm	
					*E. coli* ATCC 25922	17 mm	
					*Fusarium solani* NRC 15	17 mm	
					*S. aureus* ATCC 29213	17 mm	
					*Salmonella enterica* ATCC 25566	18 mm	
*Spirulina platensis* UTEX LB 2340	Aqueous extract	Silver	13	Spherical	*Aeromonas enteropelogenes*	6.5–14 mm	[63]
					*Aeromonas hydrophila*	6–11 mm	
					*Bacillus licheniformis*	6–12 mm	
					*Escherichia fergusonii*	8–13 mm	
					*P. aeruginosa*	0–3 mm	
					*Proteus mirabilis*	9.5–15.5 mm	
					*S. aureus*	9.5–16.5 mm	
*Oscillatoria* sp. NCCU‐369	Aqueous extract	Zinc	40–130	Spherical	*Bacillus cereus*	62.5 μg/mL	[64]
					*E. coli*	125 μg/mL	
					*K. pneumoniae*	62.5 μg/mL	
					*S. aureus*	62.5 μg/mL	
*Phormidium* sp. NCCU‐104	Aqueous extract	Copper	0–22.5	Spherical/oval	*Bacillus cereus* MCC 2243	62.5 μg/mL	[65]
					*C. albicans* MCC 1151	125 μg/mL	
					*Candida glabrata* MCC 1432	250 μg/mL	
					*E. coli* MCC 2052	250 μg/mL	
					*K. pneumoniae* KJ 938546	125 μg/mL	
					*S. aureus* MCC 2708	125 μg/mL	
*Nodosilinea nodulosa*	Aqueous extract	Cobalt	25–35	Spherical	*Aspergillus flavus*	2 μg/mL; 2–5 mm	[33]
					*Bacillus safensis*	2.31–7.31 mm	
					*B. subtilis*	2.31 µg/mL	
					*Fusarium oxysporum*	3 μg/mL; 3–7 mm	
					*P. aeruginosa*	2.34 µg/mL; 2.34–6.46 mm	
*Spirulina* sp.	Dry biomass	Silver	40–50	Quasispherical	*Acinetobacter baumannii*	50 μg/mL; 14 mm	[66]
					*E. coli*	100 μg/mL; 14 mm	
					*Streptococcus pyogenes*	80 μg/mL; 15 mm	
*Spirulina subsalsa*	Dry biomass	Silver			*Acinetobacter baumannii*	75 μg/mL; 13 mm	[66]
					*E. coli*	60 μg/mL; 12 mm	
					*Streptococcus pyogenes*	80 μg/mL; 14 mm	
*Oscillatoria princeps*	Butanol extract	Silver	100–200	Spherical	*E. coli*	60 μg/mL; 13 mm	[45]
					MRSA	100 μg/mL; 12 mm	
					*Streptococcus pyogenes*	80 μg/mL; 13 mm	
*Oscillatoria princeps*	Methanolic extract	Silver	100–200	Spherical	*E. coli*	60 μg/mL; 8.5 mm	[45]
					MRSA	100 μg/mL; 6.5 mm	
					*Streptococcus pyogenes*	80 μg/mL; 8.5 mm	
*Oscillatoria princeps*	Ethanolic extract	Silver	100–200	Spherical	*E. coli*	60 μg/mL; 10.5 mm	[45]
					MRSA	100 μg/mL; 8.5 mm	
					*Streptococcus pyogenes*	80 μg/mL; 9 mm	
*Oscillatoria princeps*	Acetone extract	Silver	100–200	Spherical	*E. coli*	60 μg/mL; 10.5 mm	[45]
					*Streptococcus pyogenes*	80 μg/mL; 9 mm	
					MRSA	100 μg/mL; 8.5 mm	
*Oscillatoria princeps*	Aqueous extract	Silver	100–200	Spherical	*E. coli*	60 μg/mL; 9 mm	[45]
					*Streptococcus pyogenes*	80 μg/mL; 8.5 mm	
					MRSA	100 μg/mL; 7 mm	
*Nostoc carneum*	Dry powdered biomass	Silver	4–22	Quasispherical	*E. coli*	12 mm	[67]
					*K. pneumoniae*	10 mm	
					*S. aureus*	9–11 mm	
*Chroococcus* sp.	Aqueous extract	Silver	11–13	Spherical	*B. subtilis*	10–15 mm	[68]
					*Micrococcus luteus*	12–21 mm	
					*P. aeruginosa*	10–14 mm	
*Spirulina maxima*	Polysaccharides	Gold	16–23	Spherical	*C. albicans* KCTC 27242	32 μg/mL	[69]
*Nostoc* sp. EA03	Aqueous extract	Zinc	50–80	Star	*E. coli* ATCC 25922	2000 μg/mL	[11]
					*P. aeruginosa* PAO1	2000 μg/mL	
					*S. aureus* ATCC 25923	64 μg/mL	
*Desertifilum* sp. EAZ03	Aqueous extract	Zinc	88.0	Rod	*E. coli* ATCC 25922	1500 μg/mL	[11]
					*P. aeruginosa* PAO1	32 μg/mL	
					*S. aureus* ATCC 25923	2000 μg/mL	
*Cyanothece‐*like coccoid unicellular cyanobacterium	Fresh biomass	Silver	23–108		*S. aureus*	2 μg/mL; 16 mm	[70]
					*Streptococcus* sp.	2 μg/mL; 18 mm	
*Arthrospira platensis*	Aqueous extract	Zinc	30–55	Spherical	*B. subtilis* ATCC 6633	12.5 μg/mL; 8–24 mm	[71]
					*C. albicans* ATCC 10231	12.5 μg/mL; 9–22 mm	
					*E. coli* ATCC 8739	12.5 μg/mL; 8–20	
					*P. aeruginosa* ATCC 9022	25 μg/mL; 9–19 mm	
					*S. aureus* ATCC 6538	50 μg/mL; 8–21 mm	
*Arthrospira platensis*	Exopolysaccharides	Gold	6–40	Spherical	*C. albicans*	0.21 mg/mL	[52]
					*Candida tropicalis*	0.085 mg/mL	
					*E. coli*	0.215 mg/mL	
					*Enterococcus faecalis*	1.315 mg/mL	
					*Salmonella enterica*	0.14 mg/mL	
					*Streptococcus mutans*	0.093 mg/mL	
*Nostoc carneum*	Phycoerythrin	Silver	7.1–26.68	Spherical	*Enterobacter aerogenes*	18 mm	[51]
					*S. aureus*	16 mm	
					*Streptococcus* sp.	18 mm	
*Spirulina platensis*	Fresh biomass	Gold	15.49–55.08	Octahedral, pentagonal, and triangular	*Aspergillus flavus* ATCC 9643	15 μg/mL	[51]
					*B. subtilis* ATCC 19659	1.95 µg/mL	
					*C. albicans* ATCC 24433	3 μg/mL	
					*Candida tropicalis* ATCC 1380	1 μg/mL	
					*K. pneumoniae* ATCC 70063	3.9 µg/mL	
					MRSA	15.63 µg/mL	
					*P. aeruginosa* ATCC 9027	7.81 µg/mL	
					*S. aureus* ATCC 25923	7.81 µg/mL	
					*Salmonella typhi* ATCC 14028	3.9 µg/mL	
*Oscillatoria* sp.	Fresh biomass	Silver	10.49–45.81	Spherical	*Aspergillus flavus* ATCC 9643	No effect	[34]
					*B. subtilis* ATCC 19659	15.63 μg/mL	
					*C. albicans* ATCC 24433	16 μg/mL	
					*Candida tropicalis* ATCC 1380	7 μg/mL	
					*K. pneumoniae* ATCC 70063	15.63 μg/Ml	
					MRSA	62.5 μg/mL	
					*P. aeruginosa* ATCC 9027	62.5 μg/mL	
					*S. aureus* ATCC 25923	31.25 μg/mL	
					*Salmonella typhi* ATCC 14028	15.63 μg/mL	
*Phormidium formosum*	Fresh biomass	Silver	1–26		Aeromonas hydrophila	2 mm	[72]
					*C. albicans*	1.9 mm	
					*E. coli*	1.7 mm	
					*Enterococcus faecalis*	1.3 mm	
					*Micrococcus luteus*	1.5 mm	
					*P. aeruginosa*	1.5 mm	
					*Proteus* spp.	1.5 mm	
					*S. aureus*	2.5 mm	
					*Salmonella* spp.	1.5 mm	
					*Serratia marcescens*	1.3 mm	
					*Vibrio* spp.	2.7 mm	
*Spirulina platensis*	Cell‐free extract	Selenium	2.8–38.9	Spherical	*S. aureus* ATCC 25923	27.5 μg/mL; 13.9 mm	[55]
					*Salmonella typhimurium* ATCC 14028	30 μg/mL; 12.7 mm	
*Spirulina*	Aqueous extract	Silver	10–200	Spherical	*Acinetobacter baumannii*	10 μg/mL	[73]
					*C. albicans*	50 μg/mL	
					*E. coli*	10 μg/mL	
					*Enterococcus faecalis*	30 μg/mL	
					*P. aeruginosa*	20 μg/mL	
					*S. aureus*	20 μg/mL	
*Desertifilum* sp.	Aqueous extract	Silver	22–40	Spherical	*Bacillus cereus* ATCC 10876	17.33 mm	[74]
					*B. subtilis* ATCC 6633	16.33 mm	
					*P. aeruginosa* ATCC 27853	15 mm	
					*Salmonella enterica*	16.67 mm	
					*Shigella flexneri*	22.67 mm	
*Nostoc* sp. Bahar M	Aqueous extract	Silver	8.5–26.4	Spherical	*K. pneumoniae*	0.9 mg/mL; 15.33 mm	[75]
*Nostoc* sp. Bahar M	Aqueous extract	Silver	8.5–26.4	Spherical	*E. coli* ATCC 25922	0.9 mg/mL; 18.6 mm	[50]
					MRSA	0.9 mg/mL; 18 mm	
					*Salmonella typhimurium* ATCC 14028	0.9 mg/mL; 14.8 mm	
					*Streptococcus mutans*	1.2 mg/mL; 14.7 mm	
*Nostoc* sp. Bahar M	Aqueous extract	Silver	8.5–26.4		*C. albicans* Ncpf 3179	1.2 mg/mL; 15.8 mm	[76]
*Desertifilum* sp. IPPAS B‐1220	Aqueous extract	Silver	4.5–26		*C. albicans* Ncpf 3179	1.2 mg/mL; 17.5 mm	[74]
*Nostoc muscorum* Lukesova 2/91	Aqueous extract	Silver	4–26	Cubic to oval	*E. coli*	15.625 μg/mL; 18.1mm	[77]
					*K. pneumoniae*	31.25 μg/mL; 19.1 mm	
					*P. aeruginosa*	31.25 μg/mL; 17.2 mm	
					*S. aureus*	3.9 μg/mL; 24.4 mm	
*O. limnetica*	Aqueous extract	Silver	3.30–17.97	Spherical	*Bacillus cereus*	20 mm	[78]
					*E. coli*	22 mm	
*Desertifilum tharense*	Exopolysaccharides	Silver	6.24–11.7	Spherical	*Bacillus cereus* ATCC 10876	9 mm	[79]
					*E. coli* O157:H7	18 mm	
					*Micrococcus luteus* ATCC 10240	9 mm	
					MRSA	17 mm	
					*P. aeruginosa* ATCC 9027	13 mm	
					*Salmonella typhimurium* ATCC 14028	13 mm	
*Phormidium ambiguum*	Exopolysaccharides	Silver	6.46–12.2	Spherical	*Bacillus cereus* ATCC 10876	12 mm	[79]
					*E. coli* O157:H7	21 mm	
					*Micrococcus luteus* ATCC 10240	9 mm	
					MRSA	25 mm	
					*P. aeruginosa* ATCC 9027	17 mm	
					*Salmonella typhimurium* ATCC 14028	14 mm	
*O. limnetica*	Aqueous extract	Iron		Trigonal rhombohedral	*Aspergillus versicolor*	27.7 µg/mL; 40–73 mm	[13]
					*B. subtilis*	14.4 µg/mL; 2.5–5 mm	
					*E. coli*	35 µg/mL; 2–4 mm	
					*P. aeruginosa*	10.7 µg/mL; 1.5–4 mm	
					*Rhizopus microsporus*	53 µg/mL; 53–75 mm	
					*S. aureus*	20 µg/mL; 3–8 mm	
*Spirulina platensis*	Aqueous extract	Silver	69.9	Spherical	*Enterococcus hirae* ATCC 9790	<5 μg/mL	[80]
					*P. aeruginosa* Gar3	<5 μg/mL	
					*S. aureus* MDC 5233	<5 μg/mL	
					*Salmonella typhimurium* MDC 1759	<5 μg/mL	
*Microcystis aeruginosa*	Aqueous extract	Silver	4–8	Spherical	*E. coli*	12.7–16.3 mm	[81]
					*S. aureus*	10.13–13.93 mm	
*Nostoc muscorum* NCCU‐442	Aqueous extract	Silver	6–45	Spherical	*S. aureus* MTCC 902	16 mm	[82]
*Nostoc linckia*	Phycobiliproteins	Silver	16.3–25.8	Spherical	*C. albicans*	3.82–10.51 mm	[83]
					*Diplococci* sp.	7.31–20.12 mm	
					*E. coli*	2.3–9.11 mm	
					*K. pneumoniae*	4.51–13.75 mm	
					*Proteus vulgaris*	3.9–15.03 mm	
					*S. aureus*	7.21–17.8 mm	
*Spirulina platensis*	Phycobiliproteins	Silver	15.1–27.4	Spherical	*C. albicans*	2.1–9.8 mm	[83]
					*Diplococci* sp.	5.82–14 mm	
					*E. coli*	1.37–4 mm	
					*K. pneumoniae*	2.95–9.10 mm	
					*Proteus vulgaris*	4.45–15.5 mm	
					*S. aureus*	6.11–12.10 mm	
*Acacia cyanophylla*	Aqueous extract	Silver	62.41–86.98	Spherical	*E. coli*	3.125–12.5 μg/mL	[84]
*Pseudanabaena/Limnothrix* sp.	Aqueous extract	Silver	6–7		*Corynebacterium glutamicum*	ND	[85]
					*E. coli*	ND	
*Synechococcus* sp.	Fresh biomass	Silver	5–10	Spherical	*B. subtilis* ATCC 6653	ND	[86]
					*E. coli* ATCC 10536	ND	
					*S. aureus* ATCC 25923	ND	
*Chroococcus turgidus*	Cell‐free extract	Silver	20.67	Spherical to oval	*E. coli* MTCC 1541	1–4 mm	[87]
					*K. pneumoniae* MTCC 3384	1–2 mm	
					*Micrococcus luteus* MTCC 1541	1–1.4 mm	
					*S. aureus* MTCC 3381	1–2 mm	
					*Salmonella paratyphi* MTCC 3220	1–4.1 mm	
*Characium typicum*	Cell‐free extract	Silver	20.67	Spherical to oval	*K. pneumoniae* MTCC 3384	1.5–2	[87]
					*E. coli* MTCC 1541	2–3.3	
					*Micrococcus luteus* MTCC 1541	1–3 mm	
					*S. aureus* MTCC 3381	1.2–3 mm	
					*Salmonella paratyphi* MTCC 3220	4.2–6.9 mm	
*Anabaena spiroides*	Aqueous extract	Gold	<80	Spherical	*Klebsiella oxytoca*	25 mg/mL; 16–19 mm	[88]
					MRSA	20 mg/mL; 16–19 mm	
					*Streptococcus pyogenes*	30 mg/mL; 16–19 mm	
*Nostoc calcicola*	Aqueous extract	Gold	20–140	Spherical	*Candida tropicalis*	250 μg/mL; 14.66 mm	[89]
					*E. coli*	500 μg/mL; 12.33 mm	
					*Klebsiella oxytoca*	13.66 mm	
					MRSA	500 μg/mL; 14.66 mm	
					*P. aeruginosa*	14.66 mm	
					*Streptococcus pyogenes*	500 μg/mL; 13.66 mm	
					*Trichophyton rubrum*	16.66 mm	
*Spirulina*	Aqueous extract	Titanium	55	Spherical	*E. coli*	62.5 μg/mL; 8–11 mm	[57]
					*Enterococcus faecalis*	31.25 μg/mL; 8–16 mm	
					MRSA	7.82 μg/mL; 17–22 mm	
					*P. aeruginosa*	31.25 μg/mL; 13–17 mm	
*Leptolyngbya* sp. L‐2	Aqueous extract	Iron	21–84	Spherical	*Alternaria alternata*	50 µg/mL; 23–48 mm	[40]
					*Botrytis cinerea*	50 µg/mL; 22–29 mm	
					*E. coli*	50 µg/mL; 5–50 mm	
					*K. pneumoniae*	50 µg/mL; 11–58 mm	
					*S. aureus*	50 µg/mL; 39–70 mm	
					*Staphylococcus coagulase* negativa	50 µg/mL; 17–65 mm	
*Nodularia haraviana*	Aqueous extract	Silver	24.1	Spherical	*B. subtilis*	4.2 μg/mL; 4.2–15.3 mm	[90]
					*E. coli*	7 μg/mL; 7–57 mm	
					*K. pneumoniae*	13 μg/mL; 13–56 mm	
					*P. aeruginosa*	4.2 μg/mL; 4.2–19.3 mm	
					*S. aureus*	33 μg/mL; 33–67 mm	
					*Staphylococcus coagulase* negativa	15 μg/mL; 15–61 mm	
*Spirulina platensis*	Aqueous extract	Silver	5–50	Spherical	*Klebsiella* sp. KC 899845	ND	[91]
					*Staphylococcus* sp. KC 688883	ND	
*Desertiflum* sp. TN‐15	Aqueous extract	Zinc	94.8	Star	*Alternaria alternata*	15 μg/mL	[39]
					*B. subtilis*	15.3 μg/mL	
					*E. coli*	41.8 μg/mL	
					*P. aeruginosa*	37.5 μg/mL	
					*S. aureus*	30.05 μg/mL	
*Aliinostoc oryzae*	Fresh biomass	Silver	50–100	Spherical	*Alternaria alternata*	12 mm	[92]
					*Botrytis cinerea*	14 mm	
					*Colletotrichum gloeosporioides*	15 mm	
					*Fusarium oxysporum*	15 mm	
					*Phytophthora capsici*	13 mm	
					*Pythium ultimum*	16 mm	
					*Rhizopus stolonifer*	14 mm	
*Arthrospira platensis*	Ethanolic extract	Silver	18–100	Spherical	*Enterococcus faecalis*	100 μg/mL; 10–16	[93]
					*P. aeruginosa*	200 μg/mL; 7–12	
					*P. mirabilis*	100 μg/mL; 7–15	
					*S. aureus*	200 μg/mL; 8.3–14 mm	
*Microcystis* sp.	Dry powdered biomass	Silver	90–130	Spherical	*Alternaria* sp.	15 mm	[44]
					*Aspergillus niger*	21 mm	
					*C. albicans*	10 mm	
					*E. coli*	No effect	
					*Fusarium* sp.	21 mm	
					*Pseudomonas* sp.	13 mm	
					*S. aureus*	9 mm	
					*Salmonella* sp.	No effect	
					*Streptococcus* sp.	No effect	
*Microcystis* sp.	Ethanolic extract	Silver	40–85	Spherical	*C. albicans*	12 mm	[44]
					*Aspergillus niger*	21 mm	
					*E. coli*	11–13 mm	
					*Fusarium* sp.	25 mm	
					*Pseudomonas* sp.	12–15 mm	
					*S. aureus*	28–29 mm	
					*Salmonella* sp.	10–13 mm	
					*Alternaria* sp.	11 mm	
					*Streptococcus* sp.	11–12 mm	
*Oxynema thaianum* ALU PBC5	Aqueous extract	Silver	8–50	Spherical	*E. coli* ALU MDR1	30–50 μg/mL	[94]
					*K. pneumoniae* ALU MDR2	30–50 μg/mL	
*Anabaena* sp.	Fresh biomass	Selenium	5–50	Spherical	*E. coli*	11 mm	[95]
					*S. aureus*	10 mm	
*Microcystis aeruginosa*	Cell‐free extract	Silver	5–45	Spherical	*E. coli* ATCC 25922	6.1 mm	[96]
					*S. aureus* ATCC 25923	5.2 mm	
*Leptolyngbya tenuis* BDU 20391	Fresh biomass	Gold	8–42	Spherical and oval	*P. aeruginosa* MTCC 424	No effect	[53]
					*S. aureus* MTCC 3160	No effect	
*Coleofasciculus chthonoplastes* BDU 61001	Fresh biomass	Gold	8–42	Spherical and oval	*P. aeruginosa* MTCC 424	No effect	[53]
					*S. aureus* MTCC 3160	No effect	
*Nostoc ellipsosporum*	Fresh biomass	Gold	8–42	Spherical and oval	*P. aeruginosa* MTCC 424	No effect	[53]
					*S. aureus* MTCC 3160	No effect	
*Anabaena* sp.	Exopolysaccharides	Silver	24.13	Irregular	*Bacillus megaterium* ATCC 13402	17 mm	[97]
					*B. subtilis* ATCC 19162	16 mm	
					*E. coli* ATCC 10836	16 mm	
					*Micrococcus luteus* ATCC 4698	20 mm	
					*P. aeruginosa* ATCC 39324	22 mm	
					*S. aureus* ATCC 29213	17 mm	
*Limnothrix* sp. 37‐2‐1	Phycocyanin extract	Silver	25.65	Spherical and elongated	*Bacillus megaterium* ATCC 13402	No effect	[97]
					*B. subtilis* ATCC 19162	No effect	
					*E. coli* ATCC 10836	No effect	
					*Micrococcus luteus* ATCC 4698	No effect	
					*P. aeruginosa* ATCC 39324	No effect	
					*S. aureus* ATCC 29213	No effect	
*Synechocystis* sp. 48‐3	Exopolysaccharides	Silver	14.64	Irregular	*Bacillus megaterium* ATCC 13402	9 mm	[97]
					*B. subtilis* ATCC 19162	No effect	
					*E. coli* ATCC 10836	10 mm	
					*Micrococcus luteus* ATCC 4698	11 mm	
					*P. aeruginosa* ATCC 39324	11 mm	
					*S. aureus* ATCC 29213	No effect	
*Lyngbya* sp. 15‐2	Exopolysaccharides	Silver			*Bacillus megaterium* ATCC 13402	20 mm	[97]
					*P. aeruginosa* ATCC 39324	10 mm	
					*B. subtilis* ATCC 19162	No effect	
					*E. coli* ATCC 10836	10 mm	
					*Micrococcus luteus* ATCC 4698	12 mm	
					*S. aureus* ATCC 29213	No effect	
*Cylindrospermopsis* sp. USC‐CRB3	Exopolysaccharides	Silver			*B. subtilis* ATCC 19162	13 mm	[97]
					*Bacillus megaterium* ATCC 13402	15 mm	
					*E. coli* ATCC 10836	12 mm	
					*Micrococcus luteus* ATCC 4698	11 mm	
					*P. aeruginosa* ATCC 39324	15 mm	
					*S. aureus* ATCC 29213	10 mm	
*Synechococcus* sp. 145‐6	Exopolysaccharides	Silver			*B. subtilis* ATCC 19162	No effect	[97]
					*Bacillus megaterium* ATCC 13402	9 mm	
					*E. coli* ATCC 10836	10 mm	
					*Micrococcus luteus* ATCC 4698	11 mm	
					*P. aeruginosa* ATCC 39324	11 mm	
					*S. aureus* ATCC 29213	No effect	
*Scytonema geitleri* HKAR‐12	Cell‐free extract	Silver	9–17	Spherical	*E. coli* strain 1	6 mm	[97]
					*E. coli* strain 2	13 mm	
					*P. aeruginosa*	8 mm	
*Anabaena iyengarii*	Phycobiliproteins	Silver		Rod	*E. coli*	9 mm	[98]
					*P. aeruginosa*	10 mm	
					*S. aureus*	17 mm	
*Oscillatoria pseudogeminata*	Phycocyanin extract	Silver	150–250		*E. coli*	8–12 mm	[99]
					*P. aeruginosa*	10–17 mm	
					*S. aureus*	18–21 mm	
*Nostoc* sp.	Fresh biomass	Silver			*E. coli* ATCC 25966	9 mm	[54]
					MRSA	2 mm	
					*P. aeruginosa* ATCC 27853	9 mm	
					*S. aureus* ATCC 25923	10 mm	
*Phormidium* sp.	Fresh biomass	Silver	5–10	Spherical	*E. coli* ATCC 25966	10 mm	[54]
					*S. aureus* ATCC 25923	9 mm	
					MRSA	11.5 mm	
					*P. aeruginosa* ATCC 27853	8 mm	
*Scytonema* sp.	Fresh biomass	Silver			*E. coli* ATCC 25966	9.5 mm	[54]
					MRSA	12 mm	
					*P. aeruginosa* ATCC 27853	9 mm	
					*S. aureus* ATCC 25923	7 mm	
*Leptolyngbya* sp. SSI24	Phycocyanin extract	Selenium	44.45–209	Spherical	*K. pneumoniae*	7 mm	[49]
					*P. aeruginosa*	6.46 mm	
					*S. aureus*	7 mm	
					*Staphylococcus epidermidis*	7 mm	
					*Staphylococcus pneumoniae*	12.74 mm	
*Chroococcus minutus*	Fresh biomass	Silver			*E. coli*	100 mg/mL; 12–16 mm	[100]
					*P. aeruginosa*	100 mg/mL; 12–16 mm	
					*S. aureus*	200 mg/mL; 12–16 mm	
*Spirulina platensis*	Aqueous extract	Copper	30–40	Spherical	*Bacillus cereus* MTCC 9017	24–30 mm	[10]
					*E. coli* MTCC 9721	19–23 mm	
					*K. pneumoniae* MTCC 9751	21–26 mm	
					*Proteus vulgaris* MTCC‐7299	27–29 mm	
					*S. aureus* MTCC 9542	23–25 mm	
					*Staphylococcus epidermidis* MTCC 2639	22–26 mm	
*Trichodesmium erythraeum*	Aqueous extract	Silver	26.5	Spherical and irregular cubical	*E. coli*	4–8 mm	[101]
					*E. coli* (amicacinaR)	5.1–10.6 mm	
					*K. pneumoniae*	4–7 mm	
					*Proteus mirabilis*	6–10 mm	
					*S. aureus*	7–11 mm	
					*S. aureus* (TetraciclinaR)	12–15.2 mm	
					*Streptococcus pneumoniae* (PenicilinaR)	11.4–18.5 mm	
					*Vibrio cholerae*	4–9 mm	
*Nostoc* sp. SI‐SN	Acetone extract	Zinc	12–26	Quasi‐spherical to hexagonal	*E. coli*	48.5 μg/mL; 15 mm	[37]
					*Klebsiella* sp.	10.06 μg/mL; 22 mm	
					*P. aeruginosa*	36.34 μg/mL; 19 mm	
					*S. aureus*	25.42 μg/mL; 20 mm	
*Spirulina platensis*	Cell‐Free Extract	Silver	30–50	Spherical	*Bacillus cereus* MTCC 9017	24.3 mm	[32]
					*E. coli* MTCC 9721	24.3 mm	
					*K. pneumoniae* MTCC 9751	25.0 mm	
					*Proteus vulgaris* MTCC 7299	31.3 mm	
					*S. aureus* MTCC 9542	31.0 mm	
					*Staphylococcus epidermidis* MTCC 2639	20.0 mm	
*Synechocystis* NCCU‐370	Aqueous extract	Titanium	16	Spherical	*Bacillus cereus* MTCC 9017	31.25 μg/mL	[12]
					*C. albicans*	125 μg/mL	
					*Candida glabrata*	250 μg/mL	
					*Candida krusei*	500 μg/mL	
					*E. coli*	31.25 μg/mL	
					*K. pneumoniae*	500 μg/mL	
*Leptolyngbya* sp. WUC 59	Cell‐free extract	Silver	20–35	Spherical	*B. subtilis*	8 mg/L	[102]
					*E. coli*	8 mg/L	
*Cylindrospermum stagnale*	Aqueous extract	Copper	12.21	Spherical	*C. albicans*	1.5 mM	[38]
					*E. coli*	0.6 mM; 11–24 mm	
					*Enterobacter cloacae*	1.7 mM; 5–17 mm	
					*K. pneumoniae*	2.4 mM; 4.5–10 mm	
					*P. aeruginosa*	2.5 mM; 4–11 mm	
*Nostoc* sp. strain HKAR‐2	Aqueous extract	Silver	51–100	Spherical	*Aspergillus niger*	3–5 mm	[103]
					*Ralstonia solanacearum*	15–25 mm	
					*Trichoderma harzianum*	4–5 mm	
					*Xanthomonas campestris*	18–23 mm	
*Nostoc* sp. strain HKAR‐2	Cell‐free extract	Gold	10–100	Spherical	*Aspergillus niger*	3–8 mm	[104]
					*Ralstonia solanacearum*	5–10 mm	
					*Trichoderma harzianum*	4–9 mm	
					*Xanthomonas campestris*	5–10 mm	
*Spirulina platensis*	Polysaccharides	Gold	2–8	Spherical	*B. subtilis*	9–20 mm	[35]
					*S. aureus*	9–18 mm	
*Lyngbya* sp.	Aqueous extract	Silver	20–50	Cuboidal, spherical and triangular	*E. coli* MTCC 443	100 mg; 16 mm	[105]
					*P. aeruginosa* MTCC 1688	300 mg; 12 mm	
					*S. aureus* MTCC 7443	200 mg; 14 mm	
*Nostoc linckia*	Aqueous extract	Silver	5–60	Spherical to oval	*Aspergillus niger*	0.4 mM	[106]
					*B. subtilis* MTCC 1427	0.37 mM	
					*C. albicans* MTCC 4748	0.61 mM	
					*E. coli* MTCC 1195	0.39 mM	
					*P. aeruginosa* MTCC 1688	0.31 mM	
					*S. aureus* MTCC 1430	0.35 mM	
					*Streptococcus pneumoniae* MTCC 2672	>1 mM	
*Oscillatoria princeps*	Aqueous extract	Silver	38	Spherical	*Aeromonas hydrophila* ATCC 7966	0.78 μg/mL; 11.8–12.4	[107]
					*Bacillus cereus*	1.56 μg/mL; 10.6–11.8	
					*B. subtilis* ATCC 6633	1.56 μg/mL; 12.2–12.4	
					*C. albicans* ATCC 10231	0.78 μg/mL (a); 12.2–13.8 mm	
					*E. coli* ATCC 25922	0.78 μg/mL; 13.4–14.6 mm	
					*Enterococcus faecalis* ATCC 29212	1.56 μg/mL (a); 11.8–12.4 mm	
					*K. pneumoniae* ATCC 13883	0.78 μg/mL; 12.2–13.6 mm	
					*P. aeruginosa* ATCC 27853	0.78 μg/mL; 12.2–12.8 mm	
					*S. aureus* ATCC 29213	0.78 μg/mL; 10.4–11.6 mm	
					*Vibrio anguillarum* ATCC 43312	0.78 μg/mL; 12.2–13.4 mm	
*Phormidium* sp.	Fresh biomass	Silver	86.38		MRSA	5–20 mm	[7]
*Synechocystis* sp.	Fresh biomass	Silver	10–35	Spherical	MRSA	16–32 mm	[108]
*Leptolyngbya JSC‐1*	Aqueous extract	Silver	5–50	Spherical	*E. coli*	25 mL; 9–21 mm	[109]
					*S. aureus*	25 mL; 7–17 mm	
*Plectonema* sp. *NCCU 204*	Aqueous extract	Silver	9–17	Spherical	*Bacillus cereus*	11–21 mm (b)	[110]
					*E. coli*	9–16 mm (b)	

IONPs generated from aqueous extracts of the strain *Oscillatoria limnetica* exhibited antibacterial activity against both Gram‐positive and Gram‐negative bacteria, with minimum inhibitory concentrations (MICs) varying from 10.7 μg/mL for *Pseudomonas aeruginosa* to 35 μg/mL for *Escherichia coli*, while the positive control presented an MIC value between 5 and 8 μg/mL [13]. Similarly, the IONPs synthesized from the aqueous extract of *Leptolyngbya* sp. L‐2 exhibited activity against *Staphylococcus aureus*, *E. coli*, *Klebsiella pneumoniae*, and coagulase‐negative *Staphylococcus*. However, their effectiveness was less pronounced, with an MIC of 50 µg/mL [40].

Regarding antifungal activity, the NPs synthesized by *Leptolyngbya* sp. L‐2 inhibited the growth of phytopathogenic *Rhizopus microsporus*, *Aspergillus versicolor*, *Alternaria alternata*, and *Botrytis cinerea*, both using agar diffusion and broth dilution methods. In the former method, the inhibition zones recorded were 75, 60, 50, and 30 mm at 50 µg/mL, respectively. In the second method, these NPs exhibited activity similar to that found in the antibacterial assay, with MICs around 50 µg/mL for all the fungi, except for *A. versicolor*, whose documented value was 27 µg/mL [13].

### 5.2 Copper Oxide NPs

Copper is essential for all living beings, playing a crucial role in various metabolic processes and acting as component of several enzymes and proteins involved in cholesterol, iron, and carbohydrate metabolism [111]. NPs derived from this element are known for their toxicity to biological organisms, creating interesting opportunities for their use in drug delivery systems for cancer treatment. Furthermore, they have been utilized in agricultural products as fertilizers and pesticides, as well as in biosensors, imaging, and antithrombotic agents [112]. In disease diagnosis, copper oxide NPs (CuONPs) have shown promise for detecting beta‐thalassemia [113].

Compared to silver NPs, CuONPs are more cost‐effective and exhibit superior physical and chemical stability. The antimicrobial properties are well‐documented in the scientific literature [114]. Similar to IONPs produced by cyanobacteria, the aqueous extract serves as the main source of reducing agents for the green synthesis of CuONP green synthesis (Table 1). However, these NPs have a smaller size range, varying from 20.7 to 40 nm. CuONPs synthesized using the aqueous extract of *Cylindrospermum stagnale* showed dose‐dependent antibacterial activity against Gram‐negative bacteria, with the highest inhibition observed at 6.5 mM. At this concentration, the NPs created inhibition zones ranging from 24 to 10 mm, with *E. coli* being the most sensitive, while *Pseudomonas aeruginosa* (11 mm) and *Klebsiella pneumoniae* (10 mm) were the most resistant. These results are reflected in the MIC values recorded for these NPs, which varied from 0.6 μg/mL for *E. coli* to nearly 2.5 μg/mL for *K. pneumoniae* and *P. aeruginosa* [38].

The use of aqueous extract was also effective for producing antimicrobial CuONPs from the strain *Phormidium* sp. NCCU‐104. The synthesized NPs exhibited greater bioactivity against Gram‐positive bacteria than against to Gram‐negative bacteria, with MIC values of 62.5 µg/mL for *Bacillus cereus* (MCC 2243) and 125 µg/mL for both *Staphylococcus aureus* (MCC 2708) and *Klebsiella pneumoniae* (KJ 938546) [65]. This difference in bioactivity can be attributed to the protective outer membrane present in Gram‐negative bacteria [115, 116].

In terms of antifungal activity, CuONPs derived from *C. stagnale* inhibited approximately 50% of the growth of *C. albicans* at a concentration of 6.5 mM [38]. In contrast, CuONPs obtained from *Phormidium* NCCU‐104 exhibited an IC_90_ of 250 µg/mL against *C. albicans* MCC 1151. At the same concentration, *Candida glabrata* experienced a 20% reduction in growth [65].

CuONPs biosynthesized from a cell‐free extract of *S. platensis*, rich in proteins, phenolics, and pigments, demonstrated significant antibacterial activity. The strongest inhibition was observed against *Proteus vulgaris*, with an inhibition zone of 28 mm, followed by *B. cereus* at 27 mm, *S. epidermidis* at 24 mm, *Staphylococcus aureus* at 23.8 mm, *Klebsiella pneumoniae* at 23.3 mm, and *E. coli* at 21.3 mm, all measured at a concentration of 500 μg/mL [10].

### 5.3 Zinc Oxide NPs

Zinc is another essential trace element that plays a crucial role in human health. It acts as a cofactor for approximately 300 metalloenzymes, influencing several physiological processes [117]. This metal is widely used in pharmaceutical formulations such as ointments and sunscreens, owing to its anti‐inflammatory properties and its ability to absorb UVA and UVB radiation [118, 119]. Zinc oxide NPs (ZnONPs) have been safely utilized in medicine and as packaging preservatives to protect food from microbial contamination due to their well‐known antimicrobial properties. The capacity of the ZnONPs to combat bacteria depends on different factors, including concentration, particle size, shape, and exposure time to the bacterial cell [120, 121].

In the biological production by cyanobacteria, a wide variety of shapes was observed, including oval, spherical, hexagonal, and flower‐like forms, with sizes ranging from 8 to 130 nm (Table 1). Aqueous extract has been the main material obtained from these photosynthetic microorganisms for the biosynthesis of ZnONPs. In the study conducted by Asif et al. [64], 30 cyanobacterial strains were used to biosynthesize ZnONPs. The smallest ZnONPs were produced using the extract of *Oscillatoria* sp., which was then selected for antimicrobial testing. Notably, the biogenic ZnONPs demonstrated significantly lower MIC values (62.5–125 μg/mL) for all tested bacterial strains compared to chemically synthesized ZnONPs, which exhibited MICs ranging from 250 to 500 μg/mL [64].

Normally, ZnONPs show greater bioactivity against Gram‐positive bacteria. In the study conducted by Shamshad and colleagues in 2024 [37], ZnONPs synthesized using the strain *Nostoc* sp. SI‐SN exhibited promising antimicrobial activity, particularly against Gram‐positive agents. The largest inhibition zone was observed against *Klebsiella* sp. (22 mm), followed by *S. aureus* (20 mm) and *P. aeruginosa* (19 mm). In contrast, *E. coli* showed a lower sensitivity, with an inhibition zone of 15 mm. The values recorded for Gram‐positive bacteria were similar to those documented for the commercial antibiotic tobramycin, which produced inhibition zones of 19 and 18 mm, respectively. MIC values confirmed the highest sensitivity for Gram‐positive microorganisms with values registered of 10.06, 25.42, 36.34, and 48.5 μg/mL for *Klebsiella* sp., *S. aureus*, *P. aeruginosa*, and *E. coli*, respectively [37]. In a similar study, ZnONPs synthesized from a different *Nostoc* strain (EA03) demonstrated comparable results. The highest MIC values were observed for *E. coli* ATCC 59222 and *P. aeruginosa* PAO1 (2000 μg/mL) and notable activity for *S. aureus* ATCC 59223 (64 μg/mL) [11].

ZnONPs synthesized from the genus *Desertifilum* also exhibited pronounced bioactivity against Gram‐positive bacteria [56]. For example, the ZnONPs produced from the aqueous extract of *Desertifilum* sp. EAZ03 strain were ineffective in inhibiting the growth of *E. coli* ATCC 59222 and *P. aeruginosa* ATCC PAO1, showing activity only at very high concentrations. In contrast, a concentration of 64 μg/mL was sufficient to completely inhibit *S. aureus* cell growth [56]. Similarly, biosynthesized ZnONPs from the *Desertifilum* sp. TN‐15 strain exhibited antimicrobial activity. The MIC for *S. aureus* was 30.05 μg/mL, while the MIC for *B. subtilis* was 15.3 μg/mL. The MIC values were higher for *E. coli* and *P. aeruginosa*, reaching values superior to 41.8 and 37.5 μg/mL, respectively [39]. Additionally, the NPs derived from the TN‐15 strain exhibited antifungal activity against *Alternaria alternata*, with an MIC of 15 μg/mL [39].

*S. aureus* and *K. pneumoniae* were among the target microorganisms evaluated for the antimicrobial activity of green‐synthesized ZnONPs derived from *Spirulina platensis*. The study also included *Streptococcus pyogenes* ATCC 19561 and *Salmonella typhi* ATCC 14028. At a concentration of 20 µg/mL, the ZnONPs exhibited greater inhibitory effects against Gram‐positive bacteria compared to Gram‐negative strains. Specifically, inhibition zones measured 17.33 mm for *S. aureus* and 14.57 mm for *S. pyogenes*, while for *S. typhi* and *K. pneumoniae*, the zones were 12.57 mm and 14.57 mm, respectively. In comparison to tetracycline at the same concentration (20 µg/mL), the ZnONPs exhibited comparable or superior activity against certain strains. The inhibition zones for tetracycline were 17.63 mm for *S. aureus*, 15.23 mm for *S. pyogenes*, 13.23 mm for *S. typhi*, and 15.07 mm for *K. pneumoniae*. The MICs were determined to be 31.25 µg/mL for *S. aureus* and 62.5 µg/mL for *K. pneumoniae* [62].

In another study by El‐belely and coworkers [71], *Arthrospira platensis* produced ZnONPs via metabolites present in the aqueous extract, such as polysaccharides, proteins, and enzymes, resulting in MIC values of 50 μg/mL for *S. aureus*, 25 μg/mL for *P. aeruginosa*, and 12.5 μg/mL for both *B. subtilis* and *E. coli*. The corresponding zones of inhibition were 8.8 mm for S. aureus and P. aeruginosa and 9.6 mm for *B. subtilis* and *E. coli* [71].

Regarding antifungal activity, ZnONPs biosynthesized for *A. platensis* showed a zone of inhibition of 21.6 mm at 200 μg/mL against *C. albicans* ATCC 10 231. However, as the concentration of NPs decreased, the inhibition zone also diminished, dropping to 13.2 mm at 50 μg/mL and to 9.6 mm at 12.5 μg/mL, indicating a direct correlation between NP concentration and antifungal efficacy [71].

### 5.4 Titanium Oxide NPs

Titanium oxide NPs (TiO_2_NPs) have been shown in vitro to be effective against a diverse range of infectious agents, which include various bacterial, fungi, algae, protozoa, viruses, prions, and microbial toxins, targeting mainly the cell membrane via oxidative stress [122]. Normally, biosynthesized TiO_2_NPs show better antibacterial activity than chemically synthesized TiO_2_NPs (Table 1) [122, 123, 124]. However, no comparative studies have been conducted to validate this trend in cyanobacteria‐derived NPs.

Aqueous extract is also the primary source of the reducing agent for producing TiO_2_NPs with antimicrobial activity using cyanobacteria. Like those produced from zinc oxide, these NPs tend to have a more expressive activity toward Gram‐positive bacteria (Table 1).

Siddiqui and coworkers [12] used aqueous extract of *Synechocystis* NCCU‐370 to synthesize TiO_2_NPs with an average size of 16 nm. These NPs demonstrated antimicrobial activity against *B. cereus* and *E. coli*, with an MIC of 31.25 μg/mL. However, for *K. pneumoniae*, a higher concentration of 500 μg/mL was needed to achieve similar antimicrobial effects [12].

In contrast, TiO_2_NPs produced from the aqueous extract of *Spirulina* had a larger average size of 55 nm and exhibited lower MIC values [57]. Among the tested bacteria, methicillin‐resistant *Staphylococcus aureus* (MRSA) was the most susceptible to TiO_2_NPs, showing an MIC value of 3.91 μg/mL. *P. aeruginosa* and *Enterococcus faecalis* displayed intermediate MIC values of 15.62 μg/mL, while *E. coli* was the most resistant, requiring a higher concentration of 31.25 μg/mL to inhibit its growth [57].

Siddiqui and coworkers [12] tested antifungal activity; the TiO_2_NPs produced by *Synechocystis* NCCU‐370 strain demonstrated a dose‐dependent effect against *C. albicans*, *C. glabrata*, and *C. tropicalis*. The MIC value for *C. albicans* was 125 μg/mL, while *C. glabrata* exhibited a higher resistance with an MIC of 500 μg/mL. *C. tropicalis* showed an intermediate sensitivity, with an MIC of 250 μg/mL [12].

### 5.5 Selenium NPs

Selenium NPs (SeNPs) possess biocompatibility, bioavailability, and low toxicity, making them promising candidates for biomedical applications [125]. Notably, those synthesized through biological processes demonstrate compatibility with human organs and tissues (Table 1) [126, 127].

Biogenic selenium NPs (B‐SeNPs) were synthesized from the cyanobacterium *Anabaena variabilis* NCCU‐441. A comparative analysis was conducted to evaluate the antimicrobial effectiveness of these biogenic NPs against chemically synthesized selenium NPs (C‐SeNPs) with respect to four bacterial strains: *S. aureus*, *E. coli*, *K. pneumoniae*, and *B. subtilis*. The results showed that the biogenic NPs were more bioactive against all tested bacteria, likely due to the presence of bioactive molecules in the materials used as reducing and stabilizing agents. Following the order above, the B‐SeNPs led to the inhibition halo in the antimicrobial testing of 10, 9, 9, and 10.5 mm, while for those chemically synthesized, the sizes were 8.8, 8.5, 6.5, and 9.2 mm [43].

A similar phenomenon was observed using the same NPs against yeast from the *C* *andida* genus at different concentrations. At 80 µg/mL, B‐SeNPs produced inhibition zones of 10.0, 12.0, and 11.0 mm for *C. glabrata*, *C. albicans*, and *Candida krusei*, respectively, while treatment with C‐SeNPs reached between 8.0 and 8.7 mm [43].

Pandey and coworkers [95] synthesized SeNPs using the wet biomass of *Anabaena* sp. PCC 7120 and tested them against *S. aureus* and *E. coli*. *S. aureus* showed higher sensitivity at 50 µg/mL, with an inhibition zone of 11 mm. For *E. coli*, the inhibition zone was 10 mm, slightly lower than the 11 mm zone produced by chloramphenicol used as positive control.

The constituents of the culture medium can influence the composition of cyanobacteria biomass, which in turn affects the properties of the resulting NPs. In a study conducted by Saad and colleagues [49], SeNPs were biosynthesized using a crude extract enriched with phycocyanin from *Leptolyngbya* sp. SSI24, grown in BG‐11 medium as well as in BG‐11 medium supplemented with 75% beet filter cake extract (BFCE). The SeNPs synthesized from the crude phycocyanin extract in BG‐11 medium exhibited inhibition zones ranging from 12.74 mm against *Streptococcus pneumoniae* to 6.45 mm against *P. aeruginosa*. In contrast, the SeNPs derived from the crude phycocyanin extract obtained from cells grown in the medium supplemented with BFCE demonstrated enhanced antibacterial activity against all tested bacteria, with inhibition zones measuring between 13.8 ± 5 mm for *S. aureus* and 26.69 ± 2.6 mm for *S. pneumoniae* [49].

The concentration of selenium precursors is another significant factor that influences the antimicrobial activity of NPs produced from this material. In a study by ElSaied et al. [55], three concentrations of sodium selenite (Na_2_SeO_3_)—1, 5, and 10 mM—were tested using *S. platensis* extract. The findings indicated that as the concentration of Na_2_SeO_3_ increased, the antimicrobial activity of SeNPs decreased. Specifically, the NPs produced from 1 mM Na_2_SeO_3_ exhibited the highest antimicrobial activity, with inhibition zones of 15.3 mm for *S. typhimurium* and 18.7 mm for *S. aureus*. The MICs for these bacteria were 22.5 and 17.5 µg/mL, respectively. In contrast, the use of 10 mM sodium selenite resulted in the lowest inhibition zones, measuring 12.7 mm for *S. typhimurium* and 13.9 mm for *S. aureus*, with MICs of 30 and 27.5 µg/mL, respectively [55].

Similar to other NPs produced using different metallic precursors, several studies have shown that SeNPs exhibit a dose‐dependent behavior. The aqueous extract from the strain *Anabaena indica* SOSA‐4 produced SeNPs with antimicrobial activity against *E. coli* MTCC443 in a dose‐dependent manner, resulting in inhibition zones of 10.03 mm at 20 µg/mL and 14.03 mm at 50 µg/mL. For *S. aureus* MTCC902, the inhibition zones measured 7.33 mm at 20 µg/mL and 12.13 mm at 50 µg/mL. In comparison, the standard antibiotic cefotaxime produced larger inhibition zones, measuring 15.03 mm for *E. coli* and 24.03 mm for *S. aureus* [61].

### 5.6 Gold NPs

Unlike the above NPs, the antimicrobial AuNPs derived from cyanobacteria have demonstrated a wider variety of sources of reducing and stabilizing agents, including protein extract, EPSs, wet biomass, aqueous extract, and cell‐free extract. The use of these cyanobacterial materials has produced AuNPs spherical in shape with sizes ranging from 2 to 140 nm (Table 1). Furthermore, this metal can be used in a variety of forms, such as NaAuCl_4_·2H_2_O (sodium gold(III) tetrachloroaurate dihydrate), Au_2_Cl_6_ (gold(III) chloride) in its anhydrous or trihydrate forms, and gold salt solution [34, 35, 52, 53, 88, 89, 104].

Different components of *Spirulina platensis* have been employed to produce AuNPs targeting bacteria and fungi, such as *B. subtilis*, *S. aureus*, and *C. albicans*. For example, AuNPs synthesized from protein extract demonstrated dose‐dependent inhibition zones of 9–20 mm against *B. subtilis*, *S. aureus* [36]. The MIC values recorded for the AuNPs synthesized from fresh biomass of *S. platensis* were 1.95 µg/mL for *B. subtilis*, 7.81 µg/mL for *S. aureus*, and 15.63 µg/mL for MRSA. Among the Gram‐negative bacteria that showed sensitivity to these nanomaterials, *S. typhi* had an MIC of 3.9 µg/mL, *K. pneumoniae* had an MIC of 7.81 µg/mL, and *P. aeruginosa* also had an MIC of 7.81 µg/mL [34]. Interestingly, *C. albicans* ATCC 24433 was very sensitive, with 3 µg/mL as the lowest effective concentration [35]. This value was comparable to the positive control Amphotericin B [34]. The polysaccharides extracted from a different *S. platensis* generated AuNPs, which are also bioactive for another *C. albicans* (KCTC 27242) but with a higher MIC (32 µg/mL). At this concentration, surface deformities were observed in the yeast cells [69].

AuNPs synthesized from EPSs of *A. platensis* also show significant antifungal activity against *C. albicans*, achieving an inhibition rate of 65.51% at 1:1 molar ratio of NaAuCl_4_:polysaccharides, with an MIC near 200 µg/mL. In comparison, *C. tropicalis* exhibited a 70% inhibition rate, with an MIC of approximately 85 µg/mL [52]. Furthermore, AuNPs from *Nostoc* sp. HKAR‐2 cell‐free extract inhibited *Aspergillus niger* and *Trichoderma harzianum*, with dose‐dependent zones ranging from 4 to 9 mm [104].

Lastly, *Anabaena spiroides*‐derived AuNPs also demonstrated antimicrobial effects, with inhibition zones of 16–19 mm against *Klebsiella oxytoca*, MRSA, and *S. pyogenes*. MIC values were 25, 20, and 30 mg/mL, respectively. Inhibition halos of 12.33 and 11.66 mm were observed for *C. tropicalis* and *Trichophyton rubrum*, respectively [88].

### 5.7 Cobalt NPs

Among the metal precursors employed in the production of NPs from cyanobacteria, cobalt has received the fewest investigations with only one report from Aslam and coworkers [128] who investigated the aqueous extract of *Nodosilinea nodulosa* as a source of reducing agents for the production of cobalt NPs (Co_3_O_4_NPs) [128]. The NPs generated showed antibacterial activity against *P. aeruginosa* and *Bacillus safensis* with an MIC near to 2.3 µg/mL. In the Kirby–Bauer antibiotic testing, for the Gram‐negative bacterium, the zones of inhibition ranged from 2.34 to 6.46 mm at concentrations of 50 and 200 µg/mL, while for the Gram‐positive bacterium, they varied from 2.31 to 7.31 mm at the corresponding concentrations. Co_3_O_4_NPs demonstrated antifungal properties, showing a zone of inhibition (ZOI) ranging from 2 mm at 50 µg/mL to 5 mm at 200 µg/mL against *Aspergillus flavus*, with an MIC of 2 µg/mL. In the case of *Fusarium oxysporum*, the ZOI ranged from 3 mm at 50 µg/mL to 7 mm at 200 µg/mL, with an MIC of 3 µg/mL (Table 1) [128].

### 5.8 Silver NPs

A variety of materials have been used to produce silver NPs (AgNPs) from cyanobacteria, including aqueous, cell‐free, and phycobiliprotein‐enriched crude extracts [82, 129, 130]. FTIR spectroscopy analysis has shown the presence of amide groups in the aqueous microalgae extract, likely derived from proteins, which can account for 65%–70% of the dry biomass weight. Regarding the cell‐free extracts, one component contributing to their ability to produce NPs is the monosubstituted amide, which is also likely derived from proteins (Table 1). This compound has a strong binding affinity to metals, which helps form a protective layer around the NPs, preventing their agglomeration and stabilizing them in the medium [131].

Several studies have explored the use of *S. platensis* as a biological agent for synthesizing AgNPs using distinct types of extracts. The AgNPs produced from aqueous extracts have shown significant antibacterial activity. At a concentration of 5 µg/mL, these NPs were effective against *S. aureus*, *Enterococcus hirae*, *P. aeruginosa*, and *S. typhimurium*, with inhibition rates ranging from 45% to 60%. In this research, these NPs demonstrated greater efficacy compared to chemically synthesized AgNPs [80]. In another study, AgNPs produced from the same material but from a different *Spirulina* species were coated with chitosan and demonstrated significant antibacterial activity. They exhibited inhibition zones measuring 20 mm against *Acinetobacter baumannii*, 17 mm against *S. aureus*, 16 mm against *E. coli*, 14 mm against *P. aeruginosa*, and 13 mm against *E. faecalis*. The MIC values for various pathogens ranged from 10 to 20 µg/mL. The inhibition zones observed in the positive control group were 58 mm for *S. aureus*, 4 mm for *E. faecalis*, and 22 mm for *C. albicans*, indicating a strong antimicrobial effect. However, no significant antifungal activity was observed against *C. albicans* [73].

A crude phycobiliprotein‐rich extract of *S. platensis* was used in the production of AgNPs with dose‐dependent antibacterial activity, resulting in inhibition zones of 15.5, 14, and 12.1 mm against *P. vulgaris*, *Diplococci* sp., and *Staphylococcus aureus* at 60 µg/mL [83]. Furthermore, cell‐free extracts from another *Spirulina* produced AgNPs with even more significant antibacterial activity, with inhibition zones of 31 mm against *P. vulgaris* and *S. aureus*. Inhibition halos for other clinically relevant strains, such as *K. pneumoniae* MTCC 9751 and *B. cereus* MTCC 9017, ranged from 24 to 25 mm at 50 μg/disk [32].

The genus *Anabaena* has also been extensively studied in the context of biosynthesized AgNPs with antimicrobial properties. As observed for *Spirulina*, different cell components have been explored for the production of NPs from these filamentous cyanobacteria. FTIR analysis showed that functional groups like hydroxyl, carbonyl, and amide (I and II) found in the phycobiliproteins from *A. variabilis* play an important role in the reduction, capping, and stabilization of produced AgNPs [41]. The NPs derived from this strain showed pronounceable antimicrobial activity with MICs ranging from 6.25 to 25 μg/mL against bacteria *P. aeruginosa*, *K. pneumoniae*, *E. coli*, and *B. cereus* [41]. *P. aeruginosa* and *E. coli* were also the target of the AgNPs synthesized by *Anabaena iyengarii* along with *S. aureus*, producing inhibition halos between 9 and 17 mm at 75 μg/mL [99].

AgNPs from *Nostoc* spp. have been synthesized using several methods, including aqueous extracts, phycoerythrin, and dry biomass. The study by El‐Naggar et al. [51] demonstrated that *Nostoc carneum* *‐*derived phycoerythrin acted as a reducing agent, yielding AgNPs that exhibited significant antibacterial activity. The inhibition zones measured 18 mm for *Streptococcus* sp. and *Enterobacter aerogenes*, 16 mm for *S. aureus*, and 21 mm for *E. coli* using 60 μL of the dispersion of 1000 μg/mL AgNPs [51]*.* In contrast, AgNPs synthesized from dry biomass showed lower antibacterial activity, with inhibition zones of 11 mm for *S. aureus* and 12 mm for *E. coli* at 15 µg/mL of AgNPs [67]*.*

Hamida et al. [77] and Husain et al. [82] conducted studies on the aqueous extracts of *Nostoc muscorum* strains, specifically Lukesova 2/91 and NCCU‐442 [77, 82]. Their findings indicate that polysaccharides, proteins, and fatty acids play a significant role in reducing and stabilizing NPs. Notably, the AgNPs produced from the *N. muscorum* Lukesova 2/91 strain, with sizes ranging from 4 to 26 nm, exhibited superior antibacterial activity against *S. aureus*, as evidenced by a 24.4 mm inhibition zone at 100 µg/mL [77]. In comparison, the AgNPs derived from the NCCU‐442 strain, which ranged from 6 to 45 nm, showed a smaller inhibition zone of 16 mm at the same AgNP concentration [82]. This data highlights the relationship between smaller particle size and enhanced antibacterial effectiveness.

The AgNPs produced from the *Nostoc* sp. Bahar_M strain not only demonstrated antibacterial activity against *E. coli* and *K. pneumoniae* but also exerted a harmful effect on *S. typhimurium* and *Streptococcus mutans*, with an inhibition zone measuring nearly 15 mm [50, 75]. However, a change to the wet biomass from the same cyanobacterium reduced the efficacy of AgNPs against these bacteria [54].

Unicellular cyanobacteria have demonstrated significant potential as a valuable source of AgNPs. In two independent studies conducted by Keskin and colleagues in 2016 [86], as well as Younis et al. in 2022 [132], the wet biomass of *Synechocystis* sp. was utilized for the synthesis of AgNPs. The antibacterial activity of these biosynthesized AgNPs was evaluated against MRSA, revealing a concentration‐dependent increase in the diameter of the inhibition zone, reaching approximately 15 mm at 15 µg/mL [132]. Furthermore, investigation of bacterial growth kinetics over a 24‐h period demonstrated that AgNPs exerted a notably stronger inhibitory effect on *B. subtilis* than on *E. coli* and *S. aureus* [86].

In the study performed with *Microcystis aeruginosa*, AgNPs exhibited inhibition zones of 6.1 mm against *E. coli* and 5.2 mm against *S. aureus* [96]. These values were lower than those reported by He and coworkers [81], who observed inhibition zones of 16.3 and 13.93 mm, respectively. Similarly, when the wet biomass from *Cyanothece* was treated with lower nitrate concentrations, smaller NPs were produced, exhibiting greater antagonistic activity compared to those obtained at higher nitrate concentrations, which resulted in larger NPs [48, 70]. The smaller NPs showed antimicrobial activity against MRSA and *Streptococcus* sp., with MICs of 2 and 1 μg/mL, respectively [70].

Similarly, *Phormidium* sp. also synthesized AgNPs with activity against multidrug‐resistant bacteria. Rashed et al. [54] reported inhibition zones of 8–11.5 mm against *P. aeruginosa*, *S. aureus*, *E. coli*, and MRSA, while Younis et al. [7] recorded zones up to 20 mm at 20 μg/mL. Notably, the combination of AgNPs with 0.5% chloramphenicol resulted in a nearly twofold increase in antibacterial activity [7].

The duration of exposure to NPs is a crucial factor affecting their antimicrobial activity. AgNPs synthesized using *Pseudanabaena*/*Limnothrix* sp. extract demonstrated strong antibacterial activity against *E. coli* and *Corynebacterium glutamicum* in a time‐ and dose‐dependent manner. The lethal concentration required to kill 50% of the test population (LC_50_) significantly decreased with increasing exposure time, reaching 7.2 μg/mL for *E. coli* and 4.5 μg/mL for *C. glutamicum* after 12 h. More than 95% of bacterial inhibition was observed with doses exceeding 25 μg/mL within 2 h. These AgNPs, which have a small average size of 6–7 nm and high monodispersity, showed greater efficacy compared to those synthesized by other cyanobacteria [85].

In addition to their bactericidal activity, several studies have investigated the antifungal potential of AgNPs. Ahamad and coworkers [41] reported MICs of 12.5 and 25 μg/mL against *C. albicans* and *C. glabrata*, respectively, using AgNPs synthesized from *A. variabilis*. Similarly, Zaki and coworkers [110] observed an MIC of 14.06 μg/mL using AgNPs from *Plectonema* sp. for the same fungal strains [110]. In studies conducted by Hamida et al. [74], AgNPs synthesized from *Desertifilum* sp. and *Nostoc* sp. produced inhibition zones measuring 15.8 and 17.5 mm for *C. albicans*, respectively [74]. Elkomy [72] reported an inhibition zone of 19 mm using AgNPs from *Phormidium formosum* [72]. Furthermore, Ismail et al. [83] documented inhibition zones of 9.8 and 10.5 mm for AgNPs synthesized from phycobiliproteins of *Spirulina platensis* and *Nostoc linckia*, respectively [83].

In the study conducted by Omar et al. [44], three synthesis methods using *Microcystis* sp. were evaluated: dry biomass, wet biomass, and ethanolic extract. The AgNPs derived from dry biomass produced an inhibition zone of 9.75 mm against *C. albicans*, comparable to the 10.5 mm inhibition zone observed with fluconazole [44]. For *Fusarium* sp., the AgNPs generated from wet biomass exhibited the greatest inhibition, with a diameter of 16.5 mm [133]. Additionally, Sidorowicz et al. [36] demonstrated that exposure to light enhances the generation of ROS and increases the antifungal activity of AgNPs synthesized from *S. platensis* in methanolic extract, with an MIC of 12 µg/mL for *C.* *krusei* [36].

## 6 Cytotoxic Properties of NPs

### 6.1 Breast Cancer

In 2022, more than 2.3 million new cases of breast cancer were diagnosed worldwide, resulting in 685,684 deaths. Projections suggest that by 2040, the global incidence of breast cancer will rise by over 40%, leading to approximately 3 million new cases each year. This highlights the urgent need for alternative treatment strategies [134].

**FIGURE 5:**
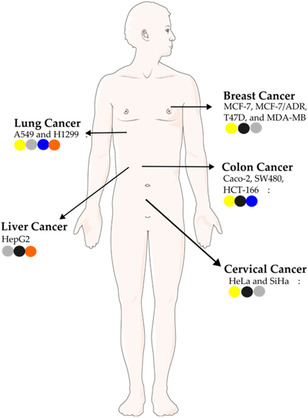
Main cell lines tested in the cytotoxic assay employing different types of NPs produced from cyanobacteria, which include CuONPs (orange circles), ZnONPs (blue circles), SeNPs (black circles), AuNPS (yellow circles), and AgNPs (silver circles).

To investigate the cytotoxic potential of cyanobacteria‐derived NPs against breast cancer, the most frequently employed cell lines include MCF‐7 (human mammary adenocarcinoma), its adriamycin‐resistant variant MCF‐7/ADR, epithelial substrain T47D, and MDA‐MB‐231, the latter being a model for more aggressive, invasive, and metastatic breast cancer [135]. Most studies have used silver, gold, and selenium as metal precursors (Figure 5). These NPs have an average size between 3.3 and 100 nm (Table 2).

**TABLE 2:** Cytotoxic activity of cyanobacteria‐mediated nanoparticles reported in the literature.

Cyanobacteria	Biocomponents	Metal precursor	Size, nm	Shape	Cell line	Tissue	Activity (IC_50_ ‐ time)	Reference
*A. variabilis* NCCU‐441	Aqueous extract	Selenium	6.4–15.8	Spherical	HepG2	Liver	96.22 μg/mL ‐ 24 h	[43]
					MCF‐7	Breast	49.69 μg/mL ‐ 24 h	
*Arthrospira indica* SOSA‐4	Aqueous extract	Selenium	4.5–14	Spherical	HEK‐293	Cervical	124.33 ‐ 24 h	[61]
					MCF‐7	Breast	14.5 ‐ 24 h	
					SiHa	Cervical	14.62 ‐ 24 h	
					SW480	Colon	18.86 ‐ 24 h	
*S. platensis*	Methanolic extract	Zinc	8–31	Hexagonal	Caco‐2	Colon	96.25 μg/mL ‐ 24 h	[62]
*S. platensis*	Soluble polysaccharides	Silver	12–15.3	Spherical	HepG2	Liver	24.5 μg/mL	[47]
					WISH	Cervical	43 μg/mL	
*S. platensis*	Ethanolic extract	Silver	7.75–18.05	Spherical	HepG2	Liver	62.1 μg/mL ‐ 24 h	[46]
					MCF‐7	Breast	56.2 μg/mL ‐ 24 h	
*Phormidium* sp. NCCU‐104	Aqueous extract	Copper	0–22.5	Spherical/oval	A549	Lung	88.3 µg/mL ‐ 24 h	[65]
					H1299	Lung	100.8 µg/mL ‐ 24 h	
*Nostoc* sp. Bahar M	Aqueous extract	Silver	30–50	Spherical	Caco‐2	Colon	150 μg/mL	[136]
*Oscillatoria salina*	Aqueous extract	Silver	100–200	Spherical	HeLa	Cervical	54.6 µL/mL ‐ 72 h	[137]
					MD‐AMB‐231	Breast	47.5 μg/mL ‐ 72 h	
*Nostoc* sp. EA03	Aqueous extract	Zinc	50–80	Star	A549	Lung	50 μg/mL ‐ 24 h	[11]
					MRC‐5	Lung	56.89 μg/mL ‐ 24 h	
*Desertifilum* sp. EAZ03	Aqueous extract	Zinc	88.0	Rod	A549	Lung	100 μg/mL ‐ 24 h	[56]
					MRC‐5	Lung	50 μg/mL ‐ 24 h	
*A. platensis*	Aqueous extract	Zinc	30–55	Spherical	Caco‐2	Colon	9.95 μg/mL ‐ 48 h	[71]
					WI‐38	Lung	53.34 μg/mL ‐ 48 h	
*A. platensis*	Exopolysaccharides	Gold	6–40	Spherical	A549	Lung	2.3 mg/mL ‐ 48 h	[52]
					Caco‐2	Colon	2.2 mg/mL ‐ 48 h	
					MCF‐7	Breast	0.5 mg/mL ‐ 48 h	
					WISH	Cervical	5.08 mg/mL ‐ 48 h	
*N. carneum*	Phycoerythrin	Silver	7.1–26.68	Spherical	MCF‐7	Breast	13.07 μg/mL ‐ 48 h	[51]
*N. linckia*	Phycoerythrin	Silver	7.1–26.68	Spherical	WI38	Lung	45.76 μg/mL ‐ 48 h	[51]
				Spherical	WISH	Cervical	52.13 μg/mL ‐ 48 h	
*S. platensis*	Fresh biomass	Gold	15.49–55.08	Octahedral, pentagonal and triangular	Caco‐2	Colon	311 μg/mL ‐ 48 h	[138]
				Octahedral, pentagonal and triangular	HeLa	Cervical	382.9 μg/mL ‐ 48 h	
				Octahedral, pentagonal and triangular	WISH	Cervical	307.31 μg/mL ‐ 48 h	
*Oscillatoria* sp.	Fresh biomass	Silver	10.49–45.81	Spherical	Caco‐2	Colon	252.83 μg/mL ‐ 48 h	[138]
				Spherical	HeLa	Cervical	286.74 μg/mL ‐ 48 h	
				Spherical	WISH	Cervical	147.77 μg/mL ‐48 h	
*Spirulina*	Aqueous extract	Silver	10–200	Spherical	Cardiomyoblast normal cell line (H9c2)	Cardiac	ND	[73]
				Spherical	Gastric cancer cell line (AGS)	Stomach	60 μg/mL ‐ 48 h	
*Desertifilum* sp.	Aqueous extract	Silver	22–40	Spherical	Caco‐2	Colon	90 μg/mL ‐ 24 h	[74]
					HepG2	Liver	32 μg/mL ‐ 24 h	
					MCF‐7	Breast	58 μg/mL ‐ 24 h	
*Nostoc* sp. Bahar M	Aqueous extract	Silver	8.5–26.4	Spherical	HCT‐116	Colon	56 μg/mL ‐ 24 h	[136]
					HepG2	Liver	80 μg/mL ‐ 24 h	
					MCF‐7	Breast	54 μg/mL ‐ 24 h	
*N. muscorum* Lukesova 2/91	Aqueous extract	Silver	4–26	Cubic to oval	A549	Lung	21.56 μg/mL	[77]
					HFs	Breast	18.78 μg/mL	
					MCF‐7	Breast	271.9 μg/mL	
					MCF7‐ADR	Breast	50.41 μg/mL	
					MD‐AMB‐231	Breast	118.1 μg/mL	
					SW480	Colon	81.42 μg/mL	
					T47D	Breast	50.02 μg/mL	
					Vero	Kidney	5.76 μg/mL	
*Phormidesmis communis* strain AB_11_10	Aqueous extract	Gold	2–28	Quasi‐spherical, triangular, and rectangular	Human osteosarcoma cell line (MG‐63)	Bone	297.5 μg/mL	[139]
					Human osteosarcoma cell line (SAOS‐2)	Bone	15.5 μg/mL	
					Vero	Kidney	861 μg/mL	
*O. limnetica*	Aqueous extract	Silver	3.30–17.97	Spherical	HCT‐116	Colon	5.37 μg/mL ‐ 48 h	[78]
					MCF‐7	Breast	6.15 μg/mL ‐ 48 h	
*S. platensis*	Phycobiliproteins	Silver	15.1–27.4	Spherical	HepG2	Liver	992 μg/mL	[83]
*C. turgidus*	Cell‐free extract	Silver	20.67	Spherical to oval	HepG2	Liver	55.78 μg/mL	[87]
					MCF‐7	Breast	40.98 μg/mL	
*N. calcicola*	Aqueous extract	Gold	20–140	Spherical	HeLa	Cervical	44.5 μL/mL ‐ 72 h	[89]
*N. calcicola*	Aqueous extract	Gold	20–140	Spherical	MD‐AMB‐231	Breast	37.3 μL/mL ‐ 72 h	[89]
*Oxynema thaianum* ALU PBC5	Aqueous extract	Silver	8–50	Spherical	A549	Lung	7.2 μg/mL ‐ 48 h	[94]
*Anabaena* sp. PCC 7120	Fresh biomass	Selenium	5–50	Spherical	HeLa	Cervical	5.5 μg/mL ‐ 24 h4.3 μg/mL ‐ 48 h2.7 μg/mL ‐ 72 h	[95]
*M. aeruginosa*	Cell‐free extract	Silver	5–45	Spherical	SiHa	Cervical	0.7 μg/mL ‐ 24 h0.8 μg/mL ‐ 48 h0.89 μg/mL ‐ 72 h	[96]
*Coleofasciculus chthonoplastes*	Fresh biomass	Gold	8–42	Spherical and oval	T‐cell acute lymphoblastic leukemia (T‐ALL)	Bone	ND	[53]
*Nostoc ellipsosporum*	Fresh biomass	Gold	8–42	Spherical and oval	T‐cell acute lymphoblastic leukemia (T‐ALL)	Bone	ND	[53]
*Scytonema geitleri* HKAR‐12	Cell‐free extract	Silver	9–17	Spherical	MCF‐7	Breast	11 μg/mL ‐ 18 h	[140]
*Leptolyngbya* sp. SSI24	Phycocyanin extract	Selenium	44.45–209	Spherical	MCF‐7	Breast	132.58 μg/mL	[49]
*T. erythraeum*	Aqueous extract	Silver	26.5	Spherical and irregular cubical	HeLa	Cervical	25 μg/mL ‐ 24 h	[101]
					MCF‐7	Breast	30 μg/mL ‐ 24 h	
					Vero	Kidney	ND	
*C. stagnale*	Aqueous extract	Copper	12.21	Spherical	HepG2	Liver	100 μg/mL ‐ 24 h50 μg/mL ‐ 48 h25 μg/mL ‐ 72 h	[38]
*Nostoc* sp. strain HKAR‐2	Aqueous extract	Silver	3.30–17.97	Spherical	MCF‐7	Breast	14 μg/mL ‐ 18 h	[103]
*Nostoc* sp. strain HKAR‐2	Cell‐free extract	Gold	10–100	Spherical	MCF‐7	Breast	250 μg/mL ‐ 24 h	[104]
*Leptolyngbya* JSC‐1	Aqueous extract	Silver	5–50	Spherical	HeLa	Cervical	160 μg/mL ‐ 24 h	[109]
*Plectonema* sp. NCCU 204	Aqueous extract	Silver	9–17	Spherical	A549	Lung	8 μg/mL ‐ 24 h	[110]

Several of *Nostoc* species have been used to biosynthesize AgNPs with cytotoxic activity against MCF‐7 breast cancer cells. The extremophile strain *Nostoc* HKAR‐2 produced AgNPs with an IC_50_ of 14 µg/mL against this cell line [103]. Additionally, AgNPs synthesized using phycoerythrin extract from *N. carneum* exhibited an IC_50_ of 13.07 µg/mL after 48 h of incubation, which, although higher than the IC_50_ of 4.81 µg/mL observed for the standard chemotherapeutic agent 5‐fluorouracil (5‐FU), suggests a comparable anticancer effect. Notably, in normal cell lines WI38 (human fetal lung fibroblast) and WISH (normal human cells), 5‐FU showed IC_50_ values of 6.68 and 5.52 µg/mL, respectively, indicating substantial cytotoxicity. In contrast, AgNPs demonstrated significantly lower toxicity toward normal cells, with IC_50_ values of 45.76 and 52.13 µg/mL. These findings underscore the lower cytotoxicity and greater selectivity of AgNPs for cancer cells compared with conventional chemotherapy [51]. However, studies have revealed considerable variations in AgNPs’ activity depending on the specific *Nostoc* strain utilized. AgNPs synthesized from the aqueous extract of *Nostoc* sp. recorded an IC_50_ of 54 µg/mL [50], while those derived from *N. muscorum* Lukesova 2/92 exhibited a significantly higher IC_50_ of 271.9 µg/mL [77].

The aqueous extract of another *N. muscorum* strain (Lukesova 2/91) also yielded AgNPs that target other breast cancer cells, including MCF‐7ADR, T47D, and MDA‐MB‐231. The IC_50_ values for these cancer cells were 50.02, 50.41, and 118.1 µg/mL, respectively. In contrast, the generated NPs demonstrated significantly higher cytotoxicity in noncancerous cell lines, such as Vero (an African green monkey kidney cell line) and normal human fibroblasts (HFs), with IC_50_ values of 5.76 and 18.78 µg/mL, respectively [47]. MDA‐MB‐231 was also sensitive to AuNPs produced from *Nostoc calcicola* with an IC_50_ of 37.3 μg/mL [89].

In a study conducted by Kuraganti and coworkers [87], the cyanobacterium *Chroococcus turgidus* was used to mediate the synthesis of AgNPs from a cell‐free extract. These AgNPs demonstrated an IC_50_ of 40.92 µg/mL against MCF‐7 cancer cells [87]. AgNPs produced from the planktonic species *Trichodesmium erythraeum* exhibited a lower IC_50_ value of 30 µg/mL [101]. Also, AgNPs synthesized by *O. limnetica* showed a dose‐dependent cytotoxicity, with an IC_50_ of 6.15 µg/mL [78].

Using gold NPs (AuNPs), the highest IC_50_ value was observed with NPs produced from EPSs derived from *A. platensis*, with a value of 500 µg/mL against MCF‐7 cells. This concentration was comparable to that found for the noncancerous epithelial WISH cell line, indicating low selectivity [52]. In contrast, biogenic NPs synthesized by *Nostoc* sp. HKAR‐2, using the same metal, exhibited half the IC_50_ value, which, however, was still higher than the IC_50_ values of AgNPs derived from cyanobacteria that target this cell line [104].

SeNPs have manifested superior activity as compared to those NPs synthesized using gold for MCF‐7 cells. The cyanobacterium *A. variabilis* NCCU‐441 produced SeNPs with an IC_50_ value of 49.69 µg/mL against MCF‐7 cells after 24 h of incubation. In contrast, chemically synthesized SeNPs exhibited a higher IC_50_ of 82.55 µg/mL, whereas the standard chemotherapy agent doxorubicin archived an IC_50_ of 0.81 µg/mL [43]. SeNPs synthesized using aqueous extract of *Arthrospira indica* SOSA‐4 reached an IC_50_ of 14.50 µg/mL, compared to 1.11 µg/mL for doxorubicin [61]. Additionally, SeNPs produced from crude phycocyanin extract of *Leptolyngbya* sp. SSI24 reduced MCF‐7 cell viability to 48.98% at 100 µg/mL, with an IC_50_ of 132.58 µg/mL [49].

### 6.2 Lung Cancer

Lung cancer is the most prevalent malignancy and remains the leading cause of cancer‐related deaths worldwide. In 2022, approximately 2.4 million new cases of lung cancer were diagnosed, resulting in around 1.8 million deaths [141]. Research has been conducted to investigate the cytotoxic effects of NPs derived from cyanobacteria on human lung carcinoma cell lines, including A549 (human alveolar basal epithelial adenocarcinoma cell line) and H1299 (nonsmall cell lung cancer cell line). Most studies have employed metal precursors such as zinc, silver, gold, and copper (Figure 5 and Table 2). The size of these NPs typically ranges from 9 to 88 nm (Table 2).

The A549 human lung carcinoma cell line exhibited dose‐dependent cytotoxicity following exposure to ZnONPs biosynthesized by cyanobacteria. ZnONPs produced using the aqueous extract of *Nostoc* sp. EA03 showed an IC_50_ of 50 μg/mL for A549 cells, whereas less than 50% of MRC‐5 (normal HF) cells were affected at the same concentration [11]. After 24 h, A549 cell viability decreased from 85.7% to 9.44%, and MRC‐5 decreased from 94.76% to 5.44% across ZnONP concentrations ranging from 10 to 200 μg/mL. Comparable results were observed with ZnONPs synthesized from *Desertifilum* sp. EAZ03, with an IC_50_ of 100 µg/mL for A549 cells [56].

Padmini and colleagues [94] reported that AgNPs synthesized from *Oxynema thaianum* ALU PBC5 inhibited the growth of A549 cells, with an IC_50_ value of 7.2 μg/mL [94]. This treatment resulted in cell shrinkage and morphological changes after 48 h. In a separate study, AgNPs derived from *Plectonema* sp. NCCU 204 exhibited an IC_50_ of 5.53 μg/mL and induced apoptotic features, including nuclear blebbing and fragmentation [110]. In contrast, AgNPs from *N. muscorum* Lukesova 2/91 had an IC_50_ of 24.79 μg/mL [77], while CuONPs synthesized from *Phormidium* sp. demonstrated an IC_50_ of 88.3 μg/mL in the same cell line.

AuNPs biosynthesized from *A. platensis* exhibited an IC_50_ value of 1.4 mg/mL, as indicated in A549. These NPs were observed to be internalized by membrane‐bound vesicles and endosomes, and they localized within the cytosol, lysosomes, and perinuclear regions [52]. Additionally, these biogenic NPs significantly influenced gene expression and demonstrated genotoxic effects in both in vitro and in vivo models, as reported [52].

CuONPs biosynthesized by *Phormidium* sp. (NCCU‐204) demonstrated an IC_50_ value of 100.8 μg/mL against the H1299 cell line. This value was significantly higher than that of the positive control, doxorubicin, which has an IC_50_ of 0.94 μg/mL. In vitro treatment with these NPs induced apoptosis and resulted in significant conformational changes [110].

### 6.3 Cervical Cancer

Cervical cancer remains one of the most common malignancies affecting women globally, with high incidence and mortality rates. In 2022, there were 660,000 new cases and 350,000 deaths worldwide [141]. Most studies investigating the cytotoxic effects of NPs synthesized from cyanobacteria derivatives have focused on their impact on HeLa (human cervical carcinoma) and SiHa (human cervical squamous cell carcinoma) cell lines. These cell lines differ in the number and type of human papillomavirus (HPV) present. HeLa cells host approximately 50 copies of HPV type 18 (HPV‐18), while SiHa cells contain 1–2 copies of HPV type 16 (HPV‐16) [142]). The two viruses are responsible for approximately 70% of cervical cancers. The NPs generated from cyanobacteria and tested against HeLa and SiHa cells are typically derived from metal precursors, such as silver, gold, and selenium, and usually have an average size ranging from 5 to 140 nm (Figure 5 and Table 2).

The cytotoxic activity of AgNPs on HeLa cells was reported by Zada and colleagues [109], using extracts from *Leptolyngbya* JSC‐1. They found that cell viability decreased as AgNPs concentration increased, dropping to 55% at the highest concentration tested (160 µg/mL), with typical features of apoptosis observed [109]. Cell extract from *T. erythraeum* resulted in AgNPs with 97% of inhibition for HeLa cells growth at a concentration of 50 µg/mL, with an IC_50(24 h)_ value of 25.0 µg/mL. AgNPs disrupt genes that regulate the cell cycle, leading to DNA damage and the induction of apoptosis in tumor cells. Several studies indicate that compounds such as alkanes, amides, and alcohols contribute to the cytotoxic effects observed with these NPs [101].

AgNPs synthesized from fresh biomass of *Oscillatoria* sp. showed an IC_50(48 h)_ of 286.74 μg/mL for HeLa cells. In contrast, the IC_50_ for normal cells (WISH) was 147.77 μg/mL [34]. Additionally, the aqueous extract of *Oscillatoria salina* showed a significantly lower IC_50_ value of 54.6 μg/mL for HeLa cells [137]. The differences in IC_50_ values can be attributed to various factors, including differences in the chemical composition of the *Oscillatoria* strains used.

The biomass of *Anabaena* sp. PCC 7120 was used to synthesize SeNPs, which demonstrated cytotoxicity with IC_50_ values of 5.5, 4.3, and 2.7 μg/mL after 24, 48, and 72 h, respectively. HeLa cells treated with the same SeNPs exhibited morphological changes, including cell shrinkage, loss of adhesion, and the formation of membrane bubbles [95].

NPsAu derived from the aqueous extract of *N. calcicola* demonstrated an IC_50_ of 44.5 μg/mL for HeLa cells after 72 h of exposure. Notably, noncancerous cell line death was minimal, even at the highest concentration of 100 mM. This suggests that these NPs selectively inhibited the proliferation of cancer cells without significantly affecting healthy cells [89]. In contrast, NPsAu synthesized from *S. platensis* exhibited an IC_50_ of 382.90 μg/mL for the same cell line and 307.31 μg/mL for WISH cells, indicating lower activity and selectivity [34].

The SiHa cell line, derived from human cervical squamous cell carcinoma, was utilized to assess the cytotoxicity of biosynthesized NPs. Silver NPs synthesized from *M. aeruginosa* inhibited the proliferation of SiHa cells in a dose‐ and time‐dependent manner. After 24 h, cell viability decreased from 90.2% at a concentration of 0.1 μg/mL to 12.3% at a concentration of 2 μg/mL. The IC_50_ values were 0.70, 0.80, and 0.89 μg/mL at 24, 48, and 72 h, respectively [96]. This cytotoxicity was linked to the generation of ROS and the induction of apoptosis, both of which occurred due to the electrostatic interaction between cationic AgNPs and the negatively charged membranes of cancer cells. Treated cells exhibited nuclear condensation and a sub‐G1 phase arrest [96].

In comparison, SeNPs synthesized using *Arthrospira indica* SOSA‐4 had lower toxicity, with IC_50_ values of 14.62 μg/mL in SiHa cells and 124.33 μg/mL in noncancerous HEK‐293 cells, indicating higher selectivity [61]. For comparison, doxorubicin showed an IC_50_ of 1.24 μg/mL. For noncancerous HEK‐293 cells (derived from human kidney), the IC_50_ was considerably higher at 124.33 μg/mL, suggesting greater SeNPs’ selectivity toward cancer cells. For reference, the IC_50_ of doxorubicin was 1.24 μg/mL [61].

### 6.4 Liver Cancer

Liver cancer accounted for an estimated 865,269 new cases and 350,000 deaths worldwide in 2022 [141]. Most studies evaluating the cytotoxic effects of NPs synthesized from cyanobacteria on liver cancer have used the HepG2 cell line. Silver, copper, and selenium are the most common metal precursors. These resulting NPs typically range in size from 4.5 to 27.4 nm (Table 2).

The HepG2 cell line, derived from human liver carcinoma, is widely used to assess the cytotoxicity of biogenic NPs. Silver NPs synthesized using ethanolic extracts of *S. platensis* demonstrated an IC_50_ value of 62.1 µg/mL [46]. In contrast, AgNPs produced from dry biomass or aqueous extracts showed limited cytotoxicity. Moreover, AgNPs synthesized from crude phycobiliprotein extracts exhibited a significantly high IC_50_ of 992 µg/mL [83]. On a more positive note, Al‐Badwy and colleagues [47] found that AgNPs created with soluble polysaccharides from *S. platensis* exhibited greater cytotoxic activity, reporting an IC_50_ of 24.5 µg/mL for HepG2 cells and 43 µg/mL for WISH cells [47]. The cytotoxicity of AgNPs in HepG2 cells was mainly attributed to the release of Ag^+^ ions, which are adsorbed onto cell surfaces and act as toxic agents [47].

AgNPs synthesized from aqueous extracts of *Desertifilum* sp. exhibited an IC_50_ of 32 µg/mL against HepG2 cells [74], while those produced by *Nostoc* sp. Bahar M showed an IC_50_ of 80 µg/mL. At this concentration, the treated cells exhibited several morphological changes, including detachment, shrinkage, rounding, reduced spreading, cluster formation, and decrease cell volume [50]. AgNPs synthesized from cell‐free extracts of *C. turgidus* reduced HepG2 cell viability to 40.9% at a concentration of 100 µg/mL. A lower concentration of 5 µg/mL yielded 63.3% cell viability, with an IC_50_ value of 55.7 µg/mL [87].

Regarding SeNPs, those synthesized by *A. variabilis NCCU‐441* exhibited an IC_50_ of 96.22 µg/mL against HepG2 cells, compared to 151.59 µg/mL for chemically synthesized counterparts. For noncancerous HEK‐293 cells, the IC_50_ values were 143.21 and 154.69 µg/mL, respectively [43].

CuONPs synthesized from *C.* *stagnale* showed concentration—and time‐dependent cytotoxicity. Growth inhibition was more pronounced after 24 h than at other time intervals. Increasing the NP concentration from 25 to 100 µg/mL also increased the inhibition of cell viability from 34% to 55.1% [38].

### 6.5 Colon Cancer

Colon cancer is estimated to have led to 660,000 new cases and 350,000 deaths worldwide in 2022 [141]. To investigate the cytotoxic effects of NP produced from cyanobacteria, the cell lines used in the study are Caco‐2 (colorectal cancer), SW480 (a human colon adenocarcinoma cell line), and HCT‐166. Most studies have utilized silver, gold, zinc, and selenium as metal precursors for these NPs, which typically have an average size between 4.5 and 26 nm (Table 2).

The Caco‐2 cell line is widely used in research on human colon cancer [143, 144, 145, 146]. AgNPs synthesized with aqueous extract of *Desertifilum* sp. exhibited an IC_50_ of 90 µg/mL [74]. In similar approach, AgNPs produced from the cell extract of *Nostoc* sp. Bahar M inhibited Caco‐2 cell proliferation in a dose‐dependent manner, with an IC_50_ of 150 µg/mL, and induced apoptosis as well as morphological alterations such as cell shrinkage, irregular shape, and plasma membrane disruption [136]. AgNPs synthesized from the fresh biomass of *Oscillatoria* sp. showed an IC_50_ of 252.83 µg/mL for Caco‐2 cells and 147.77 µg/mL for WISH cells, indicating greater cytotoxicity in noncancerous cells [34].

AuNPs derived from the fresh biomass of *S. platensis* exhibited an IC_50_ value of 311 µg/mL against Caco‐2 cells. In comparison, AuNPs produced from the EPSs exhibited an IC_50_ value of 1200 µg/mL, suggesting lower toxicity and a reduced presence of antimicrobial compounds [52]. For WISH cells, the reported IC_50_ value was 5.08 mg/mL [52].

Moreover, ZnONPs produced from the cell filtrate of *A. platensis* showed IC_50_ values of 9.95 µg/mL for Caco‐2 cells and 53.34 µg/mL for human primary neurons (WI‐83), highlighting selective cytotoxicity toward cancer cells [71]. In Vero cells, ZnONPs synthesized with the methanolic extract of *S. platensis* exhibited an IC_50_ of 96.25 µg/mL for Caco‐2 cells [62].

The cytotoxicity of AgNPs biosynthesized using the aqueous extract of the cyanobacterium *O. limnetica* was evaluated against the human colorectal carcinoma cell line HCT‐116, showing an IC_50_ of 5.369 μg/mL [78]. In comparison, silver NPs synthesized from *Nostoc* sp. Bahar M reported an IC_50_ of 56 µg/mL [50], suggesting that *O. limnetica* may contain secondary metabolites with stronger cytotoxic effects.

In SW480, Afzal et al. [61] reported that SeNPs synthesized by *Arthrospira indica* SOSA‐4 exhibited an IC_50_ near to 18.86 µg/mL after 24 h [61]. In contrast, AgNPs synthesized using an aqueous extract of *N. muscorum* Lukesova 2/91 showed an IC_50_ of 81.42 µg/mL [77].

## 7 Future Perspective and Current Challenges

Biogenic NPs are produced in an environmentally friendly way and are less toxic than those made by traditional chemical or physical methods. This makes them promising for therapeutic applications in personalized medicine, including drug delivery and disease diagnosis and imaging [147]. These biologically made NPs can aid deliver drugs to hard‐to‐reach areas in the body, preventing surgery. External tools, such as radiofrequency or magnetic fields, can guide NPs carrying medicine to specific disease sites. This approach may lower the amount of drug needed and reduce side effects from high doses. Moreover, stimulus‐responsive NP systems may enable more precise control over drug release and improve the bioavailability of various therapeutic agents [148]. Although publications on cyanobacteria‐derived NPs have increased, only a limited number of studies have examined their effects in vivo, which restricts our understanding of their potential health implications and interactions within complex biological systems. Most existing research remains focused on cellular‐level responses. This situation reflects the general scenario for most biogenic NPs, highlighting a significant gap in the current literature and posing a major challenge for their clinical translation [149]. Furthermore, the lack of standardized protocols for assessing biological activity, particularly in antimicrobial assays, restricts the comparability of results. Variations in cell number, NP concentration, assay type, and activity measurement contribute to this limitation [150]. Another significant challenge in the synthesis and characterization of biogenic NPs arises from their variable size, shape, and composition, which result from the biochemical complexity of their production. Adherence to good manufacturing practice is essential to ensure the consistency, quality, and reproducibility of the generated NPs, enabling reliable results [151].

Numerous NPs derived from cyanobacteria have demonstrated significant selectivity for tumor cell lines. Rigorous preclinical and clinical studies are required to confirm their safety for clinical application [151]. The use of biological molecules not only significantly increases interactions with microorganisms but also enhances the stability of biogenic NPs [152, 153]. This is an important attribute, as they need to maintain their original properties during transportation and storage, avoiding degradation processes such as aggregation and oxidation [151].

Multiple international regulatory agencies are formulating guidelines and recommendations to address the complexities of nanomaterial regulation, including the European Chemicals Agency in the European Union [154], the Food and Drug Administration (FDA) in the United States [155], Australia's National Industrial Chemicals in Australia [156], and the Brazilian Health Regulatory Agency (Anvisa) in Brazil [157]. In Japan, the nanomaterials are regulated by Chemical Substances Control Law, which is administered by the Ministry of Economy, Trade and Industry in collaboration with the Ministry of Health, Labour and Welfare, and the Ministry of the Environment. For therapeutic applications, it is necessary to demonstrate the effectiveness of NPs in appropriate models and to clearly describe their mechanisms of action and therapeutic targets. Additional essential information includes details on biosynthesis processes and the chemical and physical properties of the NPs [158].

The yield and biological activity of NPs synthesized by cyanobacteria depend on the cells’ physiological state, which is determined by environmental conditions. Biotic and abiotic factors such as temperature, pH, light intensity, and nutrient availability are critical in regulating NP synthesis. Minor changes in these parameters can substantially alter the physicochemical properties and bioactivity of the resulting NPs. Conversely, this metabolic flexibility can be exploited to tailor the synthesis of NPs with enhanced antiproliferative potential [159]. Therefore, standardization of culture conditions is a key strategy to improve the reproducibility of NP synthesis, with particular attention to the growth phase, which directly affects secondary metabolite production. Omics‐based approaches can also aid in mitigating the NP production fluctuation by identifying the main metabolites involved with the bioactivity, as well as their regulators [160].

The commercialization of NPs necessitates large‐scale production and high‐density cultivation of cyanobacteria in photobioreactors. This process often results in self‐shading, restricting light penetration, and producing nonuniform illumination, leading to variations in both yield and NP quality. Additionally, large‐scale cultivation increases the risk of contamination by heterotrophic bacteria and fungi, which can compromise cyanobacterial growth and introduce novel metabolites into the medium, thereby altering NP production [159]. To address the issue of nonuniform light distribution in traditional bioreactors, researchers have focused on developing novel reactor designs and mathematical models [161, 162] as well in the investigation of shading on cyanobacterial behavior [163].

## 8 Final Considerations

The exploration of biological NPs, particularly those derived from cyanobacteria, has shown promising antimicrobial activity against a wide range of bacterial and fungal pathogens, as well as cytotoxic effects against various cancer cell lines. These findings highlight the potential of cyanobacteria‐based NPs as sustainable and less toxic alternatives to conventional synthetic approaches. Their inherent biocompatibility and reduced reliance on environmentally harmful chemicals emphasize their role in advancing green nanotechnology.

Although the MIC and IC_50_ values reported for biogenic NPs are normally higher than those observed for antibiotics and anticancer agents, these comparisons should be made with caution. NPs significantly differ from small‐molecule drugs in terms of size, physicochemical properties, and mechanisms of action. Furthermore, many of these NPs are produced using biological extracts, which are complex mixtures containing a variety of secondary metabolites that can affect both stability and biological activity. The values reported for the NPs originated from cyanobacteria are in accordance with those reported for NPs obtained from other organisms, as well as those obtained through conventional methods.

Moreover, most research on NPs produced from cyanobacteria does not explore their interaction with antimicrobial drugs. In contrast, several NPs created through conventional methods have demonstrated synergy with various commercial antibiotics, effectively reducing their MIC values. Interestingly, some works here documented indicated that the biological NPs show greater activity than those synthesized chemically. This enhanced effectiveness was mainly attributed to the bioactive metabolites that are inherent in the microbial sources used for synthesis. This finding suggests a promising opportunity for developing synergistic therapies that combine biological NPs with existing drugs. In addition, many of the metals employed in the production of cyanobacterial NPs are approved by the US FDA, which may facilitate their regulatory acceptance. Importantly, biological NPs can be particularly useful in scenarios where antibiotics alone fail to work, such as when microorganisms do not respond to any antibiotics, including those on the World Health Organization's Priority Pathogens List. This also includes cases involving biofilm formation, as biofilms often show resistance to conventional antibiotics.

## Author Contributions

**Laíne Santos Ribeiro**: conceptualization, investigation, writing – original draft preparation, writing – review and editing. **Samuel Cavalcante do Amaral**: conceptualization, writing – original draft preparation, writing – review and editing, supervision. **Rhuana Valdetário Médice**: writing – review and editing, supervision. **Janaína Morone Bavini**: writing – review and editing. **Camila Manoel Crnkovic**: writing – review and editing, supervision.

## Funding

This study was supported by Fundação de Amparo à Pesquisa do Estado de São Paulo (FAPESP ‐ 2022/02872‐4), Provost of Inclusion and Belonging of the University of São Paulo (PRIP‐USP), and Pro‐Rectorate of Research and Innovation of the University of São Paulo (PRPI‐USP).

## Conflicts of Interest

The authors declare no conflicts of interest.

## Data Availability

The data that support the findings of this study are available from the corresponding author upon reasonable request.
